# Strategies for Controlling Emission Anisotropy in Lead Halide Perovskite Emitters for LED Outcoupling Enhancement

**DOI:** 10.1002/adma.202413622

**Published:** 2024-12-15

**Authors:** Tommaso Marcato, Sudhir Kumar, Chih‐Jen Shih

**Affiliations:** ^1^ Institute for Chemical and Bioengineering ETH Zürich Zürich 8093 Switzerland

**Keywords:** emission anisotropy, light‐emitting diodes, outcoupling efficiency, perovskites, transition dipole moment

## Abstract

In the last decade, momentous progress in lead halide perovskite (LHP) light‐emitting diodes (LEDs) is witnessed as their external quantum efficiency (η_ext_) has increased from 0.1 to more than 30%. Indeed, perovskite LEDs (PeLEDs), which can in principle reach 100% internal quantum efficiency as they are not limited by the spin‐statistics, are reaching their full potential and approaching the theoretical limit in terms of device efficiency. However, ≈70% to 85% of total generated photons are trapped within the devices through the dissipation pathways of the substrate, waveguide, and evanescent modes. To this end, numerous extrinsic and intrinsic light‐outcoupling strategies are studied to enhance light‐outcoupling efficiency (η_out_). At the outset, various external and internal light outcoupling techniques are reviewed with specific emphasis on emission anisotropy and its role on η_out_. In particular, the device η_ext_ can be enhanced by up to 50%, taking advantage of the increased probability for photons outcoupled to air by effectively inducing horizontally oriented emission transition dipole moments (TDM) in the perovskite emitters. The role of the TDM orientation in PeLED performance and the factors allowing its rational manipulation are reviewed extensively. Furthermore, this account presents an in‐depth discussion about the effects of the self‐assembly of LHP colloidal nanocrystals (NCs) into superlattices on the NC emission anisotropy and optical properties.

## Graphical abstract

The rise of lead halide perovskite semiconductors has enabled high‐performance LEDs with internal quantum efficiencies approaching 100%. In order to further enhance the external quantum efficiencies limited by light outcoupling effects, in this account, the strategies for reducing energy dissipation through the substrate, waveguide, and evanescent modes, with a focus on manipulating the transition dipole moment orientation are reviewed.

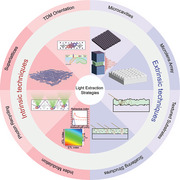

## 1 Introduction

The emergence of solution‐processed lead halide perovskite (LHP) semiconductors has generated considerable interest in realizing their exceptional optoelectronic characteristics in practical devices, including high‐performance light‐emitting diodes (LEDs).^[^ ^1^, ^2^, ^3^, ^4^, ^5^, ^6^, ^7^, ^8^ ^]^ The remarkable photoluminescence quantum efficiencies (η_PL_), narrow emission bandwidth, tunable emission wavelength, and superior charge carrier mobilities have motivated the community to apply these materials in next‐generation displays and solid‐state lighting.^[^ ^2^, ^3^, ^4^, ^5^, ^6^, ^8^, ^9^, ^10^ ^]^ In thin‐film LEDs, the external quantum efficiency (η_ext_) is one of the most important performance metrics, which is equal to the product of the internal quantum efficiency (η_int_) and the light outcoupling efficiency (η_out_) given by:

(1) $$\begin{array}{c} \;\eta_{\mathrm{ext}}=\eta_{\mathrm{int}}\times \eta_{\mathrm{out}}=\left(\gamma \times \eta_{S/T}\times \eta_{\mathrm{PL}}\right)\times \eta_{\mathrm{out}} \end{array}$$

where γ is the charge balance factor, η_S/T_ takes into account the spin character, singlet (S), or triplet (T) of the emitter excited states, and η_PL_ is the photoluminescence quantum efficiency. On the one hand, η_int_ is the efficiency for electron–hole recombination to produce photons within the LED emissive layer (EML). On the other hand, η_out_ is the light outcoupling efficiency, measuring the fraction of generated photons that are effectively extracted from the device.

In contrast to organic semiconductors, LHPs have small exciton fine splitting, so that the singlet‐triplet gap can be easily overcome by the thermal energy at room temperature, yielding a η_S/T_ of nearly unity.^[^ ^11^ ^]^ Other controlling factors, γ and η_PL_, can also be optimized to approach unity through device interface engineering and defect passivation.^[^ ^12^ ^]^ For the perovskite materials, the defect passivation involves chemically passivating the surfaces and grain boundaries to mitigate trap states that facilitate non‐radiative recombination.^[^ ^2^, ^5^, ^13^ ^]^ Numerous strategies, including core‐shell structures,^[^ ^14^, ^15^, ^16^, ^17^, ^18^ ^]^ ligand‐engineering,^[^ ^19^, ^20^, ^21^ ^]^ cation mixing,^[^ ^5^, ^22^, ^23^ ^]^ doping,^[^ ^23^, ^24^ ^]^ post‐synthetic treatment, and alloying cation sites have been explored to enhance η_PL_.^[^ ^2^, ^5^, ^25^, ^26^, ^27^, ^28^, ^29^, ^30^ ^]^ Accordingly, η_ext_ for nanocrystal (NC) PeLEDs has exceeded 30% within a decade since the first room‐temperature demonstration of PeLED.^[^ ^28^, ^29^, ^31^, ^32^ ^]^ In order to further boost η_ext_, the next step is to increase η_out_.

**Figure 1:**
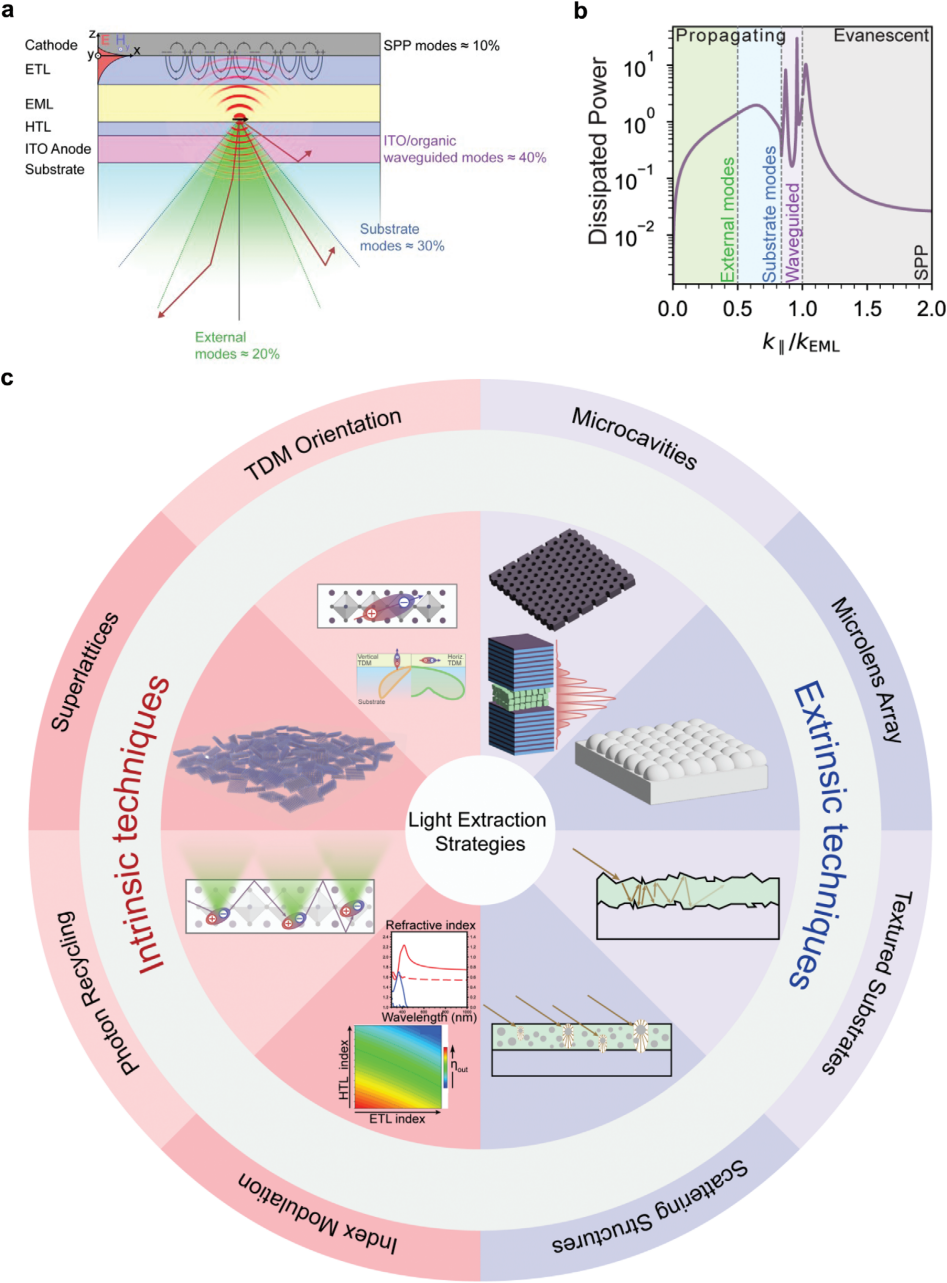
a) Schematic diagram showing a PeLED stack and the photon trapping and outcoupling modes. b) An example of the simulated dissipated power for a given radiating dipole in a PeLED as a function of the in‐plane wave vector. The regime of *k*_∥_/*k*_EML_ < 1 corresponds to the propagating modes containing the external (air) modes and the substrate or waveguided modes that are above the critical angle. For *k*_∥_/*k*_EML_ > 1, power is dissipated in the evanescent modes such as the SPPs. c) Schematic presentation for various intrinsic and extrinsic outcoupling techniques used to enhance the outcoupling efficiency of PeLEDs.

We notice that the outcoupling engineering of PeLED devices has been rather under‐explored. As shown in **Figure** **1**a,b, η_out_ is an optical factor corresponding to the ratio of photons coupled out from the device's thin‐film stack into the air mode to the total number of photons generated through the radiative decay of excited states. In first approximation, only photons emitted in directions within the critical angle $\theta_{c}=\sin^{-1}\left(\frac{1}{n}\right)$, where n is the refractive index of the emissive layer, can escape into the air, leading to a solid angle for light extraction Ω  =  2π(1 −  cos θ_*c*_) ≈ 2π/2*n* ^2^ which is a small fraction of the total 2π available for a planar device with a reflective electrode. As a consequence, the η_out_ of PeLEDs can be estimated as η_out_ = 1/(2n^2^), where n is the refractive index of the emissive layers.^[^ ^33^, ^34^ ^]^ Accordingly, for the bulk LHP EML, given *n*  ≈  2.0 −2.7, one could estimate η_out_ of ≈ 15%.^[^ ^12^, ^35^, ^36^, ^37^, ^38^, ^39^ ^]^

The remaining fraction of internally generated photons are dissipated through either propagating but trapped modes (see Figure 1b), including the substrate or waveguided modes, or the evanescent modes, typically the surface plasmon polaritons (SPPs) at the electron transport layer (ETL)/cathode interface. Both extrinsic and intrinsic light outcoupling techniques can be used to enhance η_out_. Thanks to the development of organic LED (OLED) technology, several extrinsic light outcoupling techniques, including microlens arrays (MLAs), hemispherical lenses (HSL), textured substrates, and refractive index matching layers have been proposed.^[^ ^40^, ^41^, ^42^ ^]^ In particular, the MLA approach has been extensively investigated to increase the η_out_ and η_ext_ by alleviating the substrate mode losses.^[^ ^43^ ^]^ However, these approaches are relatively difficult to integrate into commercial products, as they often demand complicated and expensive fabrication and could affect the inherent emission characteristics.

On the other hand, as shown in Figure 1c, in terms of LHP LEDs, the intrinsic light outcoupling techniques include: i) the optimization of optical properties and device thin‐film configuration, such as tuning the thickness and refractive index of LHP EML, ii) morphology control of LHP EML for the generation of anisotropic emission, iii) low refractive‐index or anisotropic carrier transport materials, and iv) design of multilayer structures for the minimization of waveguide, plasmonic, and absorption losses. In particular, a notable technique involves engineering the intrinsic emission anisotropy of excitonic states in semiconductor thin films. Indeed, the generation of an exciton, which is a bound state of an electron and a hole, corresponds to a transition polarization mediated by the transition dipole moment (TDM). The TDM directly determines the strength of light–matter coupling, the radiative lifetime, and the polarization and angular characteristics of the radiation generated upon exciton recombination. Specifically, the emission generated from exciton recombination can be described with a classical oscillating point dipole oriented along its TDM direction. The radiation is therefore preferentially emitted toward the direction orthogonal to the TDM vector. As such, if one could make the TDM vectors preferentially oriented in parallel to the substrate plane of an LED, a larger fraction of radiation is directed within the critical angle. Considering an EML of finite thickness, the effect of an ensemble of generated excitons can be characterized by a single parameter, Θ_IP_, which quantifies the fraction of TDM vectors parallel to the substrate surface and equals to the ensemble average of the in‐plane (IP) projection of the TDM. A preferentially horizontal TDM orientation can substantially enhance η_out_ by up to 50%, without adding fabrication complexity. Clearly, the characterization for the emission TDM in LHP films is crucial for understanding the probability of radiation power coupled to air upon photo‐ or electrical excitations.

The TDM orientation‐induced emission anisotropy has been extensively explored in OLEDs and quantum‐dot LEDs (QLEDs) based on colloidal cadmium selenide (CdSe) nanoplatelets (NPLs). The spontaneous emission in organic molecules involves highly localized excitons, which determines a strong correlation between the TDM and molecular orientations that helps enable rational molecular design and thin‐film processing.^[^ ^44^ ^]^ On the other hand, although excitons in inorganic semiconductor crystals originate from extended band edge states of complex 3D symmetry, which typically results in isotropic TDM orientation, excitons in CdSe NPLs have completely in‐plane emission (Θ_IP_ = 1).^[^ ^45^, ^46^, ^47^ ^]^ Indeed, the emission in quantum‐confined zinc‐blende NPLs originates exclusively from the heavy‐holes valence band states. Due to their mixed *p_x_* and *p_y_* symmetry, the bright plane coincides with the NPLs plane.^[^ ^45^ ^]^ The lessons we learned from organic and QD emitters, however, can only partially be applied to LHPs. It is required to have a systematic understanding of the origin of emission anisotropy, including factors affecting the TDM orientation in inorganic and organic–inorganic hybrid LHPs.

Here, we will review various extrinsic and intrinsic light extraction techniques applied in PeLEDs. We will discuss in‐depth and compare different intrinsic light extraction techniques and their synergy with other approaches (Figure 1c). Thereafter, we exclusively focus on different aspects of emission anisotropy in the LHP films, including the effects of long‐range ordering in self‐assembled NC superlattices. Finally, we underlined the most suitable experimental techniques to characterize the emission anisotropy and TDM orientation in the LHP films. The insights presented here are anticipated to contribute to the development of efficient and scalable PeLEDs, paving the way for their integration into advanced display and lighting technologies as well as other applications.

## 2 Extrinsic Light Outcoupling Techniques

The extrinsic light outcoupling techniques introduce outcoupling structures that are not usually part of a conventional LED stack, including microlens arrays, airside scattering structures, periodic resonant structures, and photonic crystal or index matching structures. Within this category, we also include optical microcavities as they typically require the fabrication of high‐quality mirrors, such as the distributed Bragg reflectors (DBRs). We will quickly review the recent applications to PeLEDs as these topics have been thoroughly documented elsewhere.^[^ ^43^, ^48^, ^49^ ^]^

### 2.1 Microlens Arrays and Hemispherical Lenses

The microlens arrays and hemispherical lenses (HSL) are among the most explored light‐extraction techniques to couple out the photons from substrate modes. The MLAs can be made from different materials, such as polyethylene terephthalate (PET), polystyrene (PS), polyvinyl alcohol (PVA), polydimethylsiloxane (PDMS), and silica (SiO_2_).^[^ ^42^, ^49^ ^]^ These MLAs and HSLs could reduce the incident angle below the critical angle at the substrate/air interface taking advantage of multiple reflections, thereby suppressing the total internal reflection (TIR) and boosting η_out_ considerably.^[^ ^50^ ^]^ For example, by attaching a hemispherical lens on the air side of the glass substrate, Kim et al. reported a maximum η_ext_ of 45.5% and current efficiency (η_CE_) of 205 cd A^−1^, >90% higher than their control devices.^[^ ^50^ ^]^ Together with their optical simulations, they suggested that the maximum η_out_ for their optimal PeLEDs before and after HSL attachment reached 30.2% and 55.61%, respectively. Likewise, Bai et al. demonstrated the highest η_ext_ of 55.67% in green‐emitting PeLEDs in the HSL‐attached devices, which is 81% higher than the control device.^[^ ^32^ ^]^ These recent reports endorse the fact that MLAs and HSLs are very effective for the enhancement of light extraction from the substrate modes.

### 2.2 Textured Substrates

**Figure 2:**
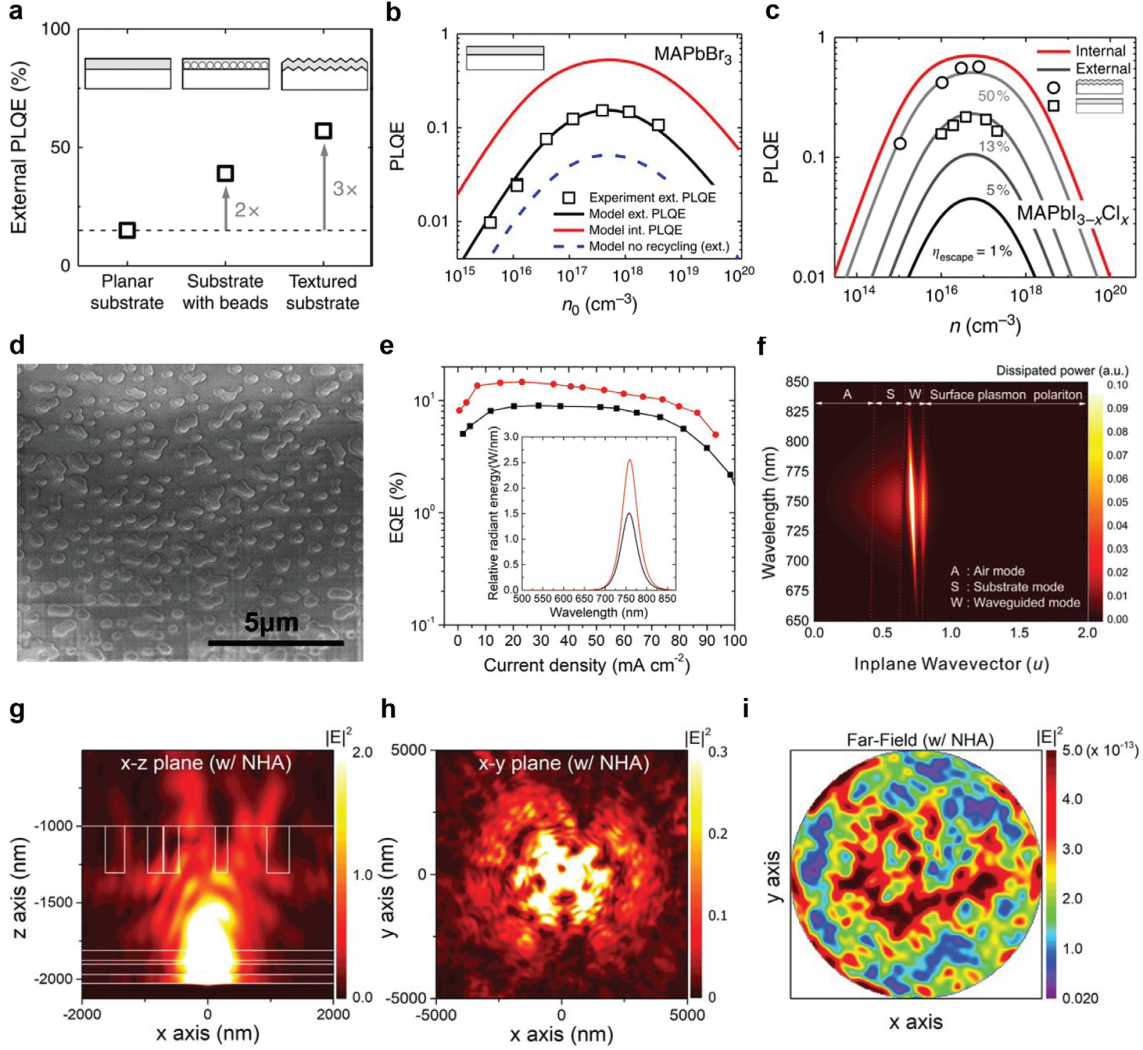
Extrinsic light outcoupling techniques. a) Effects of textured substrate surfaces on external η_PL_ for MAPbI_3−x_Cl_x_ thin films. b) External η_PL_ for MAPbBr_3_ films as a function of initial carrier density *n*_0_. c) External and internal η_PL_ of mixed halide perovskite, MAPb_3−x_Cl_x_, as a function of initial carrier density *n*_0_. Reproduced with permission.^[^ ^51^ ^]^ Copyright 2016 from Springer Nature. d) Scanning electron microscope (SEM) image showing the nanohole arrays (NHAs) between glass substrate and ITO layers. e) Comparison of characterized device η_ext_ for planar and NHA substrates. Inset: Normalized electroluminescent (EL) spectra for the two devices. f) Optical simulations for the dissipated power distribution, illustrating the energy dissipation pathways in a PeLED device. g,h) The electronic field intensity distribution in the g) *x*–*z* plane and h) *x*–*y* plane. i) Far‐field intensity distribution outside the glass substrate. Reproduced with permission.^[^ ^52^ ^]^ Copyright 2019, Wiley‐VCH.

Textured substrate is another conventional technique to mitigate energy dissipation through the substrate modes in OLEDs.^[^ ^40^ ^]^ This technique has also been exploited in perovskite solar cells to collect more light for the enhancement of absorption in the perovskite layer. Richter et al. have demonstrated approximately four‐fold enhancement in the externally characterized η_PL_ of perovskite films by substituting a planar glass substrate with the textured counterpart.^[^ ^51^ ^]^ The resulting external η_PL_ is very close to the internal η_PL_ because of enhanced light outcoupling (**Figure** **2**a–c). Hence, it is a technique worth exploring to boost the performance of PeLEDs.

### 2.3 Air‐Side Scattering Structures

In order to suppress the energy dissipation through the waveguide modes, resulting from the high refractive index of EML, Jeon et al. inserted a randomly distributed high‐index nanohole array (NHA) embedded in a silicon nitride (SiN) layer sandwiched between ITO electrode and glass substrate (Figure 2d).^[^ ^52^ ^]^ The device η_out_ is considerably improved, as reflected by the increased η_ext_ from 8.9% to 14.6% in MAPbI_3_ nanocrystallites‐based devices, after using the NHA embedded SiN layer (Figure 2e). Although the device η_ext_ was increased as compared to a control device, the efficiencies remained on the lower side, owing to the strong waveguide losses (Figure 2f). The role of NHA‐embedded SiN layer on η_out_ was further assessed using the 3D finite‐difference time‐domain (3D‐FDTD) method, and the Fourier analysis was further carried out to understand the arbitrariness of the NHA (Figure 2g–i). Their calculations suggest that an increase in the refractive index of the high‐index matrix amplifies the E‐field intensity, highlighting the effect of the high‐index matrix on η_out_. The MAPbI_3_ nano crystallites also exhibited anisotropic emission with Θ_IP_ value of 71%.^[^ ^52^ ^]^ In addition, the η_out_ of LED devices has been enhanced by introducing patterned nanostructures in the substrate, which strongly suppress the losses from the substrate mode.^[^ ^53^, ^54^, ^55^ ^]^ Cai et al. reported transparent PeLEDs with an η_ext_ of 13.6% by introducing a highly transparent silver‐coated glass substrate with nano‐pillar structure (Ag‐GPS) possessing scattering characteristics.^[^ ^53^ ^]^ To boost the *η*_out_ of the top‐emitting PeLED devices, a highly reflective planer Ag film has been deposited at the bottom side of the glass that diminishes the microcavity effect by modulating the cavity length. However, the planer Ag film generated the surface plasmon and waveguided losses, which are curtailed by replacing it with the Ag‐GPS film.

### 2.4 Manipulating Resonant Modes and Optical Cavity Design

Another category of extrinsic approaches for the enhancement of *η*_out_ is using the optical microcavity effect.^[^ ^56^, ^57^, ^58^, ^59^, ^60^ ^]^ Consider a Fabry–Pérot cavity consisting of an emissive layer of refractive index, *n*, sandwiched between two planar metallic or dielectric mirrors, in which the emitted photons are reflected back and forth between the mirrors. The constructive interference takes place when the round‐trip phase ϕ follows $2\phi =\frac{4\pi }{\lambda }nL\cos\theta =2m\pi$, where *L* is the length of the cavity, λ is the photon wavelength, and θ is the photon angle. Accordingly, the cavity is characterized by a set of discrete longitudinal standing modes of the cavity order $m_{c}=\mathrm{round}\left(\frac{2nL}{\lambda }\right)$ and the equally spaced free‐spectral difference $\Delta \lambda =\frac{2nL\cos\theta }{m(m+1)}$. The enhancement of the *η*_out_ reaches the maximum, when the cavity length is designed to make only one mode fall below the critical angle, yielding $\eta_{\mathrm{out}}=\frac{1}{m_{c}}$.

An important factor for the design is the emission bandwidth, and its relation to the cavity quality factor. The quality factor is a function of the end mirror's reflectivity and determines the bandwidth of its optical resonance. Therefore, the PeLED device architectures can be typically distinguished by the quality factor of their optical cavity resulting in “weak” or “strong” microcavities. The first regime is characterized by a quasi‐monochromatic source, where the cavity width is larger than the source linewidth. It is the most suitable for LEDs as it features increased extraction, brightness, and low chromatic aberrations. Most bottom‐emission PeLEDs are within the weak microcavity regime as the typically used transparent ITO anode has high transmittance.^[^ ^61^ ^]^ On the other hand, in the strong microcavity limit, where the cavity response is narrower than the source bandwidth, the radiation pattern becomes largely angular‐dependent.^[^ ^62^ ^]^ Although the source bandwidth is narrowed, different wavelengths are emitted in different angles as separate lobes leading to strong chromatic variation with respect to the viewing angle. This is typically the case in top‐emission devices with metal contacts, where the design of the microcavity becomes crucial to device performance. An additional and undesirable regime corresponds to a very large source bandwidth, such that both brightness and outcoupling enhancement are considerably lost, as a large part of the emission spectrum cannot be efficiently extracted.

**Figure 3:**
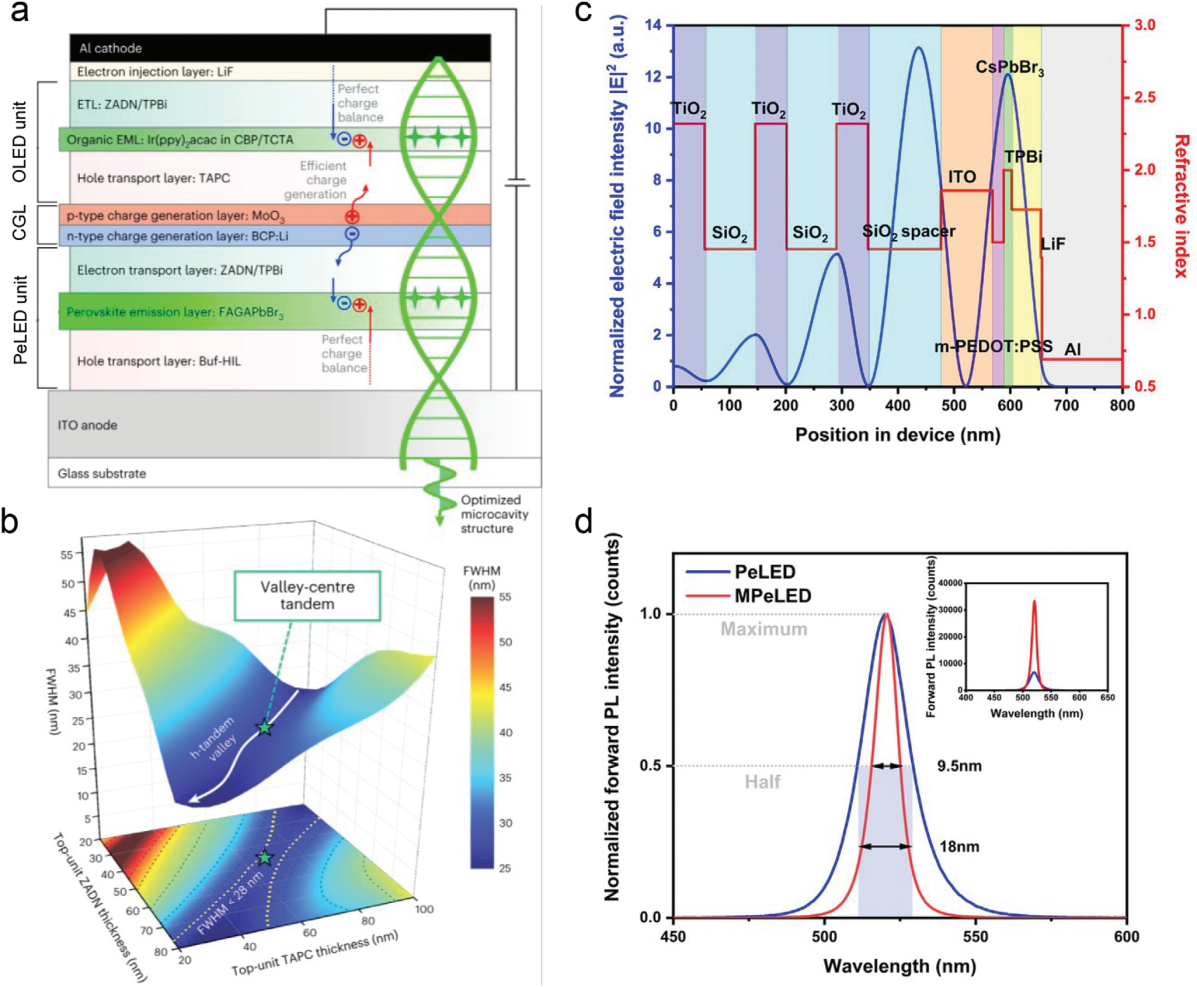
a) Schematic diagram for an organic‐LHP tandem device with an optimized microcavity structure. b) Optimization of spectral narrowing on the microcavity parameter landscape in PeLED tandem devices. Reproduced with permission.^[^ ^63^ ^]^ Copyright 2024 from Springer Nature. c) Electric field and refractive index profiles across a microcavity PeLED. d) Effect of spectral narrowing in a microcavity PeLED. Reproduced with permission.^[^ ^62^ ^]^ Copyright 2022, Wiley‐VCH.

The concept of optical microcavity has been exploited to boost the light extraction efficiency of the planer tandem PeLEDs. The tandem hybrid LEDs fabricated based on a charge generation layer (CGL) could achieve a higher η_ext_ by combining the efficiencies of individual light‐emitting units. Lee et al. reported η_ext_ of 37% with color pure green emission and a narrow spectral width of 27.3 nm in the two units of LHP and organic hybrid tandem LEDs (**Figure** **3**a,b).^[^ ^63^ ^]^ Later on, Kong et al. further confirmed the role of optical microcavity structure demonstrating a maximum η_ext_ of 43.42% in the perovskite‐organic two‐unit hybrid tandem LEDs.^[^ ^64^ ^]^ The optical microcavity effect has been recently utilized to improve the η_out_ of top‐emitting PeLEDs based on microcrystalline quasi‐2D FAPbI_3_.^[^ ^65^ ^]^ The schematic layer sequence and corresponding refractive indices of a bottom‐emission microcavity PeLED are depicted in Figure 3c.^[^ ^62^ ^]^ A high η_ext_ of above 20% has been demonstrated together with a strong spectral narrowing in a bottom emission green PeLED (Figure 3d).^[^ ^62^ ^]^

## 3 Intrinsic Light Outcoupling Techniques

### 3.1 Refractive Indices Modulation

**Figure 4:**
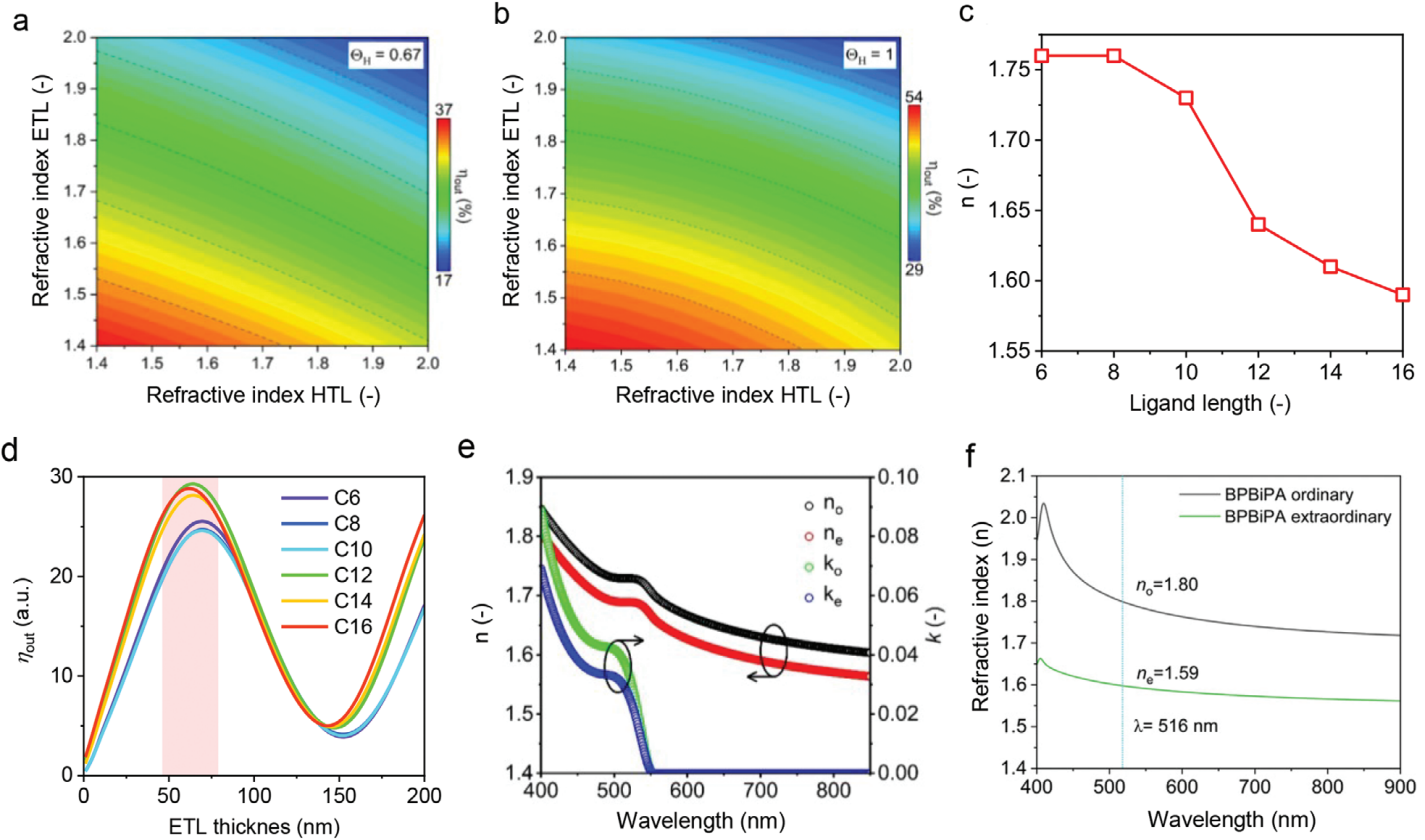
Effects of thin‐film refractive indices on the LED η_out_. a,b) Calculated η_out_ as a function of ETL and HTL refractive indices for EML of isotropic TDM orientation (Θ_IP_ = 0.67, a) and completely in‐plane TDM orientation (Θ_IP_ = 1, b). c) Thin‐film refractive indices of the LHP NCs as a function of ligand alkyl chain length from 6 to 16 carbon atoms. d) Calculated η_out_ as a function of ETL thickness for ligand alkyl chain length from 6 to 16 carbon atoms. Reproduced with permission.^[^ ^19^ ^]^ Copyright 2019 from the American Chemical Society. e) The characterized extraordinary and ordinary refractive indices (*n_e_*,*k_e_*) and (*n*_0_,*k*_0_) for FA_0.5_MA_0.5_PbBr_3_ anisotropic NC films. Reproduced with permission.^[^ ^73^ ^]^ Copyright 2022 from Springer Nature. f) Extraordinary and ordinary refractive indices (*n_e_* and *n*_0_) of an ETL material, BPBiPA. Reproduced with permission.^[^ ^74^ ^]^ Copyright 2024, Wiley‐VCH.

In PeLEDs, device η_out_ is often suppressed because of perovskite's high refractive index, *n* = 2.0–2.7,^[^ ^12^, ^35^, ^36^, ^37^, ^38^, ^39^, ^66^ ^]^ which is considerably higher than the neighboring organic carrier transporting and injection layers. Usually, most organic carrier transporting materials have a refractive index of 1.8 ± 0.15.^[^ ^67^, ^68^ ^]^ The index mismatch at interfaces creates an optical barrier for photon emission, which causes unfavorable waveguide losses. As briefly discussed earlier, the LED η_out_ is directly correlated with the refractive index of the emission layer as well as carrier transport layers (**Figure** **4**a,b).^[^ ^69^ ^]^ Indeed, the waveguide losses can account for up to 40% fraction of the total photons generated in the devices (Figure 1a). Recently, three major strategies have been used to address the interlayer waveguide losses: i) the modulation of the refractive index of the perovskite NC EML through choosing appropriate ligands or by tailoring the composition of LHPs,^[^ ^19^ ^]^ ii) synthesis or selection of low‐refractive‐index carrier‐transport layers.^[^ ^67^, ^70^, ^71^, ^72^ ^]^ iii) co‐evaporation of low‐refractive‐index fluoropolymers, such as poly‐perfluoro‐3‐butenylvinylether (PBVE), with the molecular carrier transport materials.^[^ ^68^ ^]^

Regarding the PeLEDs based on perovskite NCs, our group reported that the refractive indices of LHP NC films can be actively modulated by varying the alkyl chain length of alkylamine‐type passivating ligands.^[^ ^19^, ^20^ ^]^ Upon increasing the alkyl chain length from six to sixteen carbon atoms, the refractive indices of mixed cation FA_0.5_MA_0.5_PbBr_3_ NCs gradually decreased from 1.78 to 1.58, thereby increasing η_out_ (Figure 4c,d).^[^ ^19^ ^]^ The change in the refractive indices of LHP NC films results from the reduction of their dielectric contrast (ε), which decreases with increasing alkyl chain length, as $n=\sqrt{\varepsilon }$. The downside is that the LHP charge‐carrier mobility greatly reduces with hydrophobic alkyl chain length.^[^ ^19^ ^]^ It appears desirable to find optimal ligands that can passivate the NC defects and decrease the refractive index, without compromising the carrier mobility. Hassan et al. reported a significantly high η_ext_ of up to 32.4% in the color pure red PeLED devices using the mixed halide MAPb(I_1−x_Br_x_)_3_ NCs capped with ethylenediaminetetraacetic acid (EDTA) and reduced l‐glutathione ligands.^[^ ^69^ ^]^ The capping ligand pair not only stabilizes the NCs phase but also results in a low refractive index, (*n* = 1.82 at 620 nm) of mixed halide NCs.

The development of high‐efficiency OLED in the past decades has proven that the device η_out_ can be sufficiently increased by using low‐index HTL and ETL.^[^ ^75^, ^76^ ^]^ In PeLEDs, for example, upon introducing the low‐index HTL, X‐F6‐TAPC (*n* = 1.54),^[^ ^70^ ^]^ and ETL, 3TPYMB (*n* = 1.66),^[^ ^71^ ^]^ the LED η_out_ can be increased from 19 to 32% (Figure 4a,b).^[^ ^73^ ^]^ Upon decreasing the refractive index of perovskite NC films (Figure 4c,e) and replacing the high‐index B3PYMPM (*n* = 1.84)^[^ ^72^ ^]^ ETL with 3TPYMB, η_ext_ of PeLED devices increased from 8.69 to 24.96%.^[^ ^73^ ^]^ Besides, the organic electronic community has long recognized that the Θ_IP_ of emission TDM in the carrier transporting layers, HTL and ETL, is also a critical factor for improving the η_out_ of OLED devices.^[^ ^75^, ^77^ ^]^ For example, Sun et al. also demonstrated peak η_ext_ of >30% in the green PeLED devices (Figure 4f) by using a LHP NC film as EML and an anisotropic low‐index ETL, 9,10‐bis(4‐(2‐phenyl‐1Hbenzo[d]imidazole‐1‐yl)phenyl) anthracene (BPBiPA), that also displays preferably in‐plane TDM orientation (Θ_IP_ = 93%).^[^ ^74^ ^]^ We consider that the development of low‐index carrier transport materials with high in‐plane molecular orientation is a critical factor in boosting the η_out_ of the PeLEDs.

### 3.2 Modulation in Emission Layer Morphology and Thickness

**Figure 5:**
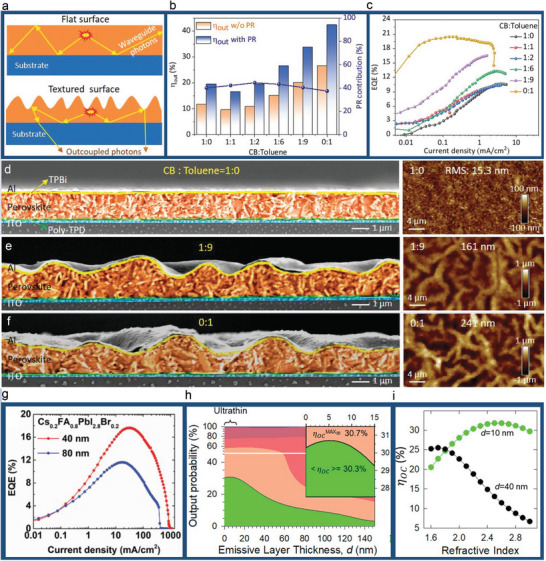
Effects of perovskite EML morphology and thickness. a) Schematic diagram comparing light outcoupling mechanisms between the planar (top) and textured (bottom) perovskite films. b) External and internal η_PL_ of textured LHP films. c) Effect of chlorobenzene (CB):toluene ratio on η_ext_ values of PeLEDs based on the textured LHP EMLs. The morphology of textured EMLs prepared using different CB:toluene ratios of d) 1:0., e) 1:9, and f) 0:1. Reproduced with permission.^[^ ^79^ ^]^ Copyright 2022, Wiley‐VCH. g) Effect of Cs_0.2_FA_0.8_PbI_2.8_Br_0.2_ EML thickness on the η_ext_ of PeLEDs. Reproduced with permission.^[^ ^36^ ^]^ Copyright 2019, Wiley‐VCH. h) Contour plot showing optically simulated η_out_ as a function of the EML thickness. Inset: magnification for the regime of ultrathin EMLs. i) Simulated η_out_ as a function of refractive index for typical (40 nm, black) and ultrathin (10 nm, green) EMLs. Reproduced with permission.^[^ ^80^ ^]^ Copyright 2023 from the American Chemical Society.

In addition to the refractive index, the EML thickness and morphology also play an important role in η_out_ enhancement by suppressing the internal losses through the waveguide modes (**Figure** **5**a–f). For example, a report has shown that the morphology of perovskite EML films can be modulated by introducing multifunctional aromatic additives, such as 2‐(4‐(methylsulfonyl)phenyl) ethylamine, without hampering the intrinsic characteristics of the bulk crystalline phase.^[^ ^78^ ^]^ These additives not only suppress the non‐radiative recombination but also alleviate interfacial quenching by covering the inter‐grain space.

Note that the EML thickness also plays a role in η_out_ as it directly influences the waveguide modes. For example, by reducing the thickness of Cs_0.2_FA_0.8_PbI_2.8_Br_0.2_ EML from 80 nm to 35–40 nm, Zhao et al. reported enhanced η_out_ and η_ext_ of more than 30% in PeLED devices (Figure 5g).^[^ ^36^ ^]^ Indeed, as discussed earlier, by changing the perovskite EML from 1.5 µm‐thick planar to thin textured films through altering the dwell time of antisolvents and photon recycling effect (Figure 5d–f),^[^ ^79^ ^]^ η_out_ can be increased from 19.5% to 42.3%. The same scenario also applies to the perovskite NC films. Very recently, Wan et al. reported η_ext_ of up to 26.7% in green perovskite NC devices, which they attribute to substantially enhanced η_out_ of >30% in ultrathin 10 nm CsPbBr_3_ NC emissive layer (Figure 5h).^[^ ^80^ ^]^ The calculated η_out_ as a function of the EML refractive index suggested that light outcoupling in 10 nm EML behaves very differently as compared to the typical 40 nm counterpart. Instead of a monotonic decrease, there exists an optimal refractive index (Figure 5i),^[^ ^80^ ^]^ which can be designed to coincide with the perovskite EML thickness. In other words, ultrathin EML could be a feasible approach to enable high‐efficiency devices based on the high‐index evaporated PeLEDs.

### 3.3 Internal Waveguide Engineering with Nano‐Patterned Interfaces

**Figure 6:**
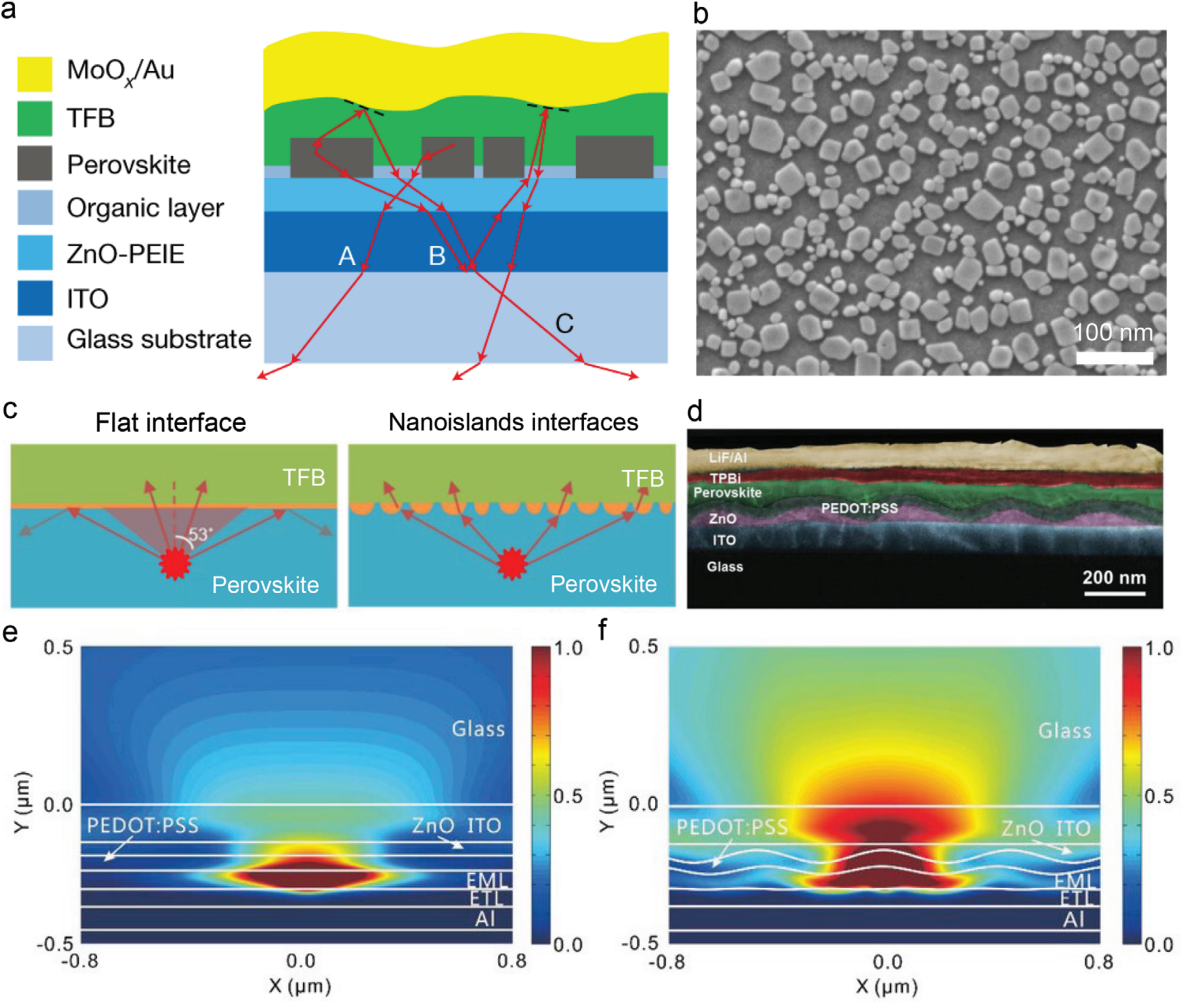
Effect of nanostructured LiF layer on PeLED efficiencies. a) Schematic diagram showing device architecture for the illustration of light extraction mechanism. b) SEM image of the perovskite film with sub‐micrometer nano‐islands. Scale bar, 1 µm. Reproduced with permission.^[^ ^81^ ^]^ Copyright 2018 from Springer Nature. c) Schematic illustration of light propagation in the PeLEDs having flat (left) and nanoisland interfaces (right). Reproduced with permission.^[^ ^82^ ^]^ Copyright 2024 from the American Chemical Society. d) Cross‐sectional SEM image for the nanostructured CsPbBr_3_‐based PeLED device. FDTD simulations illustrating light propagation in PeLEDs having flat e) and nano‐island f) EMLs. Reproduced with permission.^[^ ^83^ ^]^ Copyright 2019, Wiley‐VCH.

The interfaces between the LHP EML and transport layers could also influence the outcoupling efficiencies. Controlling the EML morphology and structuring the adjacent dielectric layers are promising strategies to improve η_out_. For example, recent literature has demonstrated that by controlling the EML morphology having sub‐micrometer structures together with optimal additive mixing (**Figure** **6**a,b), the PeLED η_out_ improved to 30%.^[^ ^79^, ^81^ ^]^ The resulting device showed an η_ext_ of 21.8% at room temperature because of low η_PL_ (≈70%) of the FAPbI_3_ EML.^[^ ^81^ ^]^ A higher η_ext_ of up to 30% was recorded at low temperatures (6K), approaching near‐unity η_int_ thanks to the reduction of non‐radiative losses.^[^ ^81^ ^]^ The same authors suggested that sub‐micrometer EML structures can be made by controlling the following factors: i) the modulation of precursor concentrations, ii) introducing molecular additives for the enhancement of η_PL_ and radiative lifetime by suppressing the defect density, and iii) the ratio of A‐ and B‐site cation precursors, FAI and PbI_2_, which also reduces the defect density. Yang et al. took advantage of nanostructured EML to demonstrate pure‐red‐emitting PeLEDs of high η_ext_ up to 21.8%, resulting from enhanced light outcoupling at interfaces between LiF and quasi‐2D (PEA_5_NMA_5_)_0.2_CsPb_2_I_7_ perovskite EML.^[^ ^82^ ^]^ Given the large dipole moment (6.3D) and the strong electronegativity of fluorine ions, the LiF is an excellent choice for mixed solvent etching that produces nano‐patterns above EML and HTL, poly(9,9‐dioctylfluorene‐alt‐N‐(4‐sec‐butylphenyl)‐diphenylamine) (TFB) layer (Figure 6c). The nanostructured low‐index LiF layer could substantially boost device performance, allowing more light to escape from the internal waveguide modes through scattering.^[^ ^80^ ^]^ Shen et al. have also demonstrated an η_ext_ of 20.3% by using bioinspired moth‐eye nanostructures (MEN) between the LHP EML and ITO.^[^ ^83^ ^]^ They have used a soft imprinting technique to form the MEN structures of ZnO and PEDOT:PSS layers (Figure 6d). Through finite‐difference‐time‐domain (FDTD) simulation, they have also demonstrated that the near‐field intensity distribution is notably enhanced in the device with MEN structures compared to planar counterparts (Figure 6e,f). This further affirms the contribution of MEN structures in high η_out_.

### 3.4 Plasmonic Nanostructures

**Figure 7:**
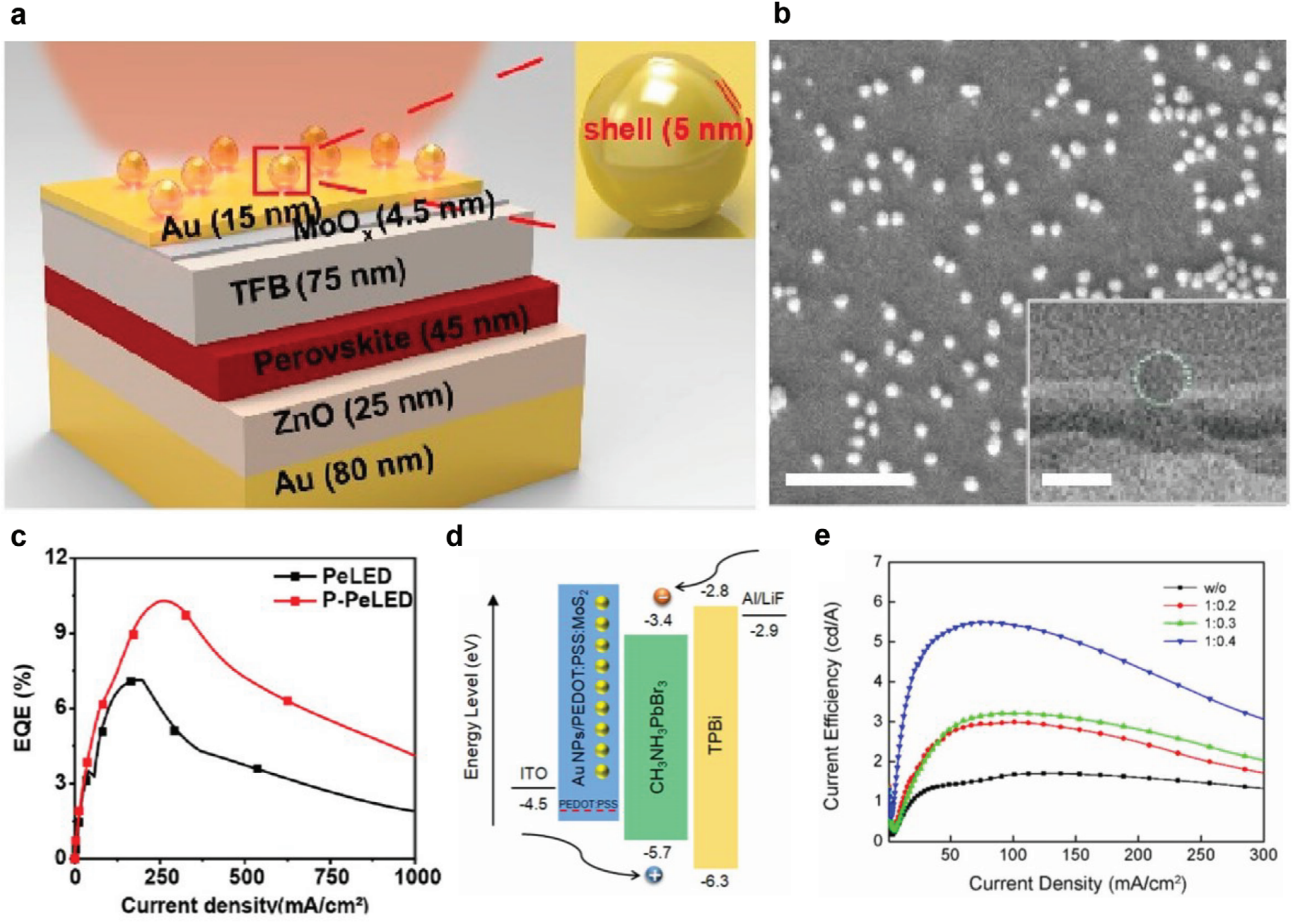
a) Schematic illustration for the top‐emission plasmonic‐ (P‐) PeLED taking advantage of plasmonic nanoantenas on the top Au electrode layer. b) The top‐view SEM images for the core‐shell Au nanoparticles. c) Comparison of η_ext_ for conventional PeLED and P‐PeLED as a function of current density. Reproduced with permission.^[^ ^92^ ^]^ Copyright 2023 from the American Chemical Society. d) Schematic energy level diagram illustrating device architecture of bottom‐emission P‐PeLED based on the Au NPs and MoS_2_‐doped PEDOT:PSS hole injection layer. e) Comparison of current efficiency, for control device with undoped PEDOT:PSS and Au:MoS_2_ doped PEDOT:PSS with varying MoS_2_ doping content, as a function of current density. Reprinted with permission.^[^ ^94^ ^]^ Copyright 2021 from IOP Publishing Ltd.

The surface plasmon (SP) modes in the PeLEDs could account for up to 60% of the total energy dissipated resulting from the coupling of photons from emission layers and the oscillating electrons from the metal cathode.^[^ ^66^, ^84^, ^85^ ^]^ The SP modes contain: i) localized surface plasmon (LSP) and ii) surface plasmon polariton (SPP). Several strategies have been proposed to enhance η_out_ by mitigating the SP losses (**Figure** **7**).^[^ ^36^, ^85^ ^]^ The most straightforward approach is to increase the distance between the emission layer and the metal cathode by using an optimally thick ETL,^[^ ^86^, ^87^, ^88^, ^89^ ^]^ which, however, considerably compromises the internal waveguide losses in the device. Alternatively, recent reports have suggested that doping gold and silver nanoparticles in the hole injection and transport layers results in a strong η_out_ and η_ext_ improvement.^[^ ^90^, ^91^, ^92^, ^93^ ^]^ For example, Zhang et al. reported an up to 1.5 times improvement by embedding plasmonic nanostructures comprising the core–shell gold nanoparticles on the top metal electrode (Figure 7a–c).^[^ ^92^ ^]^ Notably, the plasmonic structure offers non‐destructive fabrication. Sun and co‐workers reported an 223% improvement in the current efficiency of the PeLED device by doping the MoS_2_ in the PEDOT:PSS hole injection layer (Figure 7d,e).^[^ ^94^ ^]^ It is also noteworthy that the SP loss can be suppressed by choosing an anisotropic emitter with a preferentially in‐plane TDM orientation.^[^ ^95^, ^96^ ^]^

### 3.5 Horizontal Emission TDM Orientation

**Figure 8:**
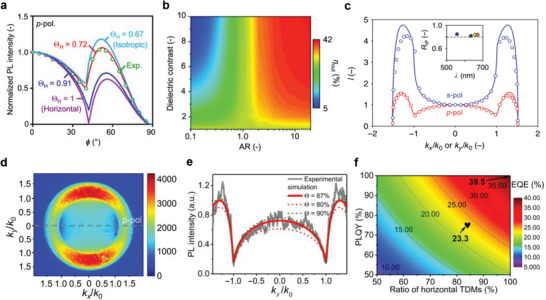
a) Effects of the in‐plane dipole ratio, Θ_IP_ (Θ_H_ in the figure), on the angular‐dependent PL intensity considering a thin FA_0.5_MA_0.5_PbBr_3_ anisotropic NC EML layer deposited on glass. b) Effects of dielectric contrast and NC aspect ratio (AR) on η_out_. Reproduced with permission.^[^ ^73^ ^]^ Copyright 2022 from Springer Nature. c) The *p*‐polarized (*p*‐pol: red) and *s*‐polarized (*s*‐pol: blue) PL intensity profiles as a function of *k_x_*/*k_0_* and *k_y_*/*k_0_*, respectively, of anion exchange green sample. Inset: Extracted thin‐film Θ_IP_ values as a function of emission wavelength. Reproduced with permission.^[^ ^20^ ^]^ Copyright 2020 from Springer Nature. d) Back focal plane (BFP) image of a *k*‐space radiation pattern of in situ growth of LHP film consisting of face‐on monolayer NPLs. e) The *p*‐polarized BFP linecut in comparison with the theoretical fitting to estimate the TDM orientation^.^ f) Effect of PLQY (η_PL_) and emission TDM orientation (Θ) on η_out_. Reproduced with permission.^[^ ^98^ ^]^ Copyright 2021 from American Association for the Advancement of Science.

As discussed earlier, the TDM is the vector quantity that describes the transition polarization of excitons and their radiative recombination. The excitons emit photons that have a cosine angular distribution with their maximum orientation perpendicular to the TDM. Therefore, in a uniform optical environment, the alignment of ensemble TDMs yields photons emitted anisotropically. Randomly oriented TDMs, corresponding to Θ_IP_ = 0.67, yields isotropic emission, while horizontally oriented TDM of Θ_IP_, also known as Θ_H_, up to 86% (experimental) and 91% (from optical simulations) in the LHP NPL films have been demonstrated to effectively outcouple more photons from the PeLED devices.^[^ ^20^, ^97^, ^98^ ^]^ As illustrated in **Figure** **8**a,b, both surrounding dielectric contrasts and the aspect ratio of LHP NCs can strongly influence the Θ_IP_ and η_out_.^[^ ^19^, ^73^, ^99^ ^]^ We have also demonstrated that the lateral alignment of anisotropic NCs (ANCs) and NPLs in the LHP films also enhanced the Θ_IP_.^[^ ^73^, ^100^ ^]^ The long‐range lateral alignment and face‐on orientation of ANCs and NPLs in the LHP films are critical to maximize Θ_IP_. To precisely control the face‐on or edge‐on orientation of LHP NPLs, Ye et al. utilized several solvents with varying vapor pressures to control the thin‐film morphology by obtaining the periodic superlattice of NPLs (more details see Section 5.3).^[^ ^101^ ^]^ Note that the extracted Θ_IP_ values of LHP NCs remained intact after changing their anion composition (Figure 8c).^[^ ^20^ ^]^ Optical simulations suggest that the dielectric contrast and aspect ratio of NCs could substantially boost η_out_ to ≈50%.^[^ ^73^ ^]^ Very recently, several reports have demonstrated enhanced emission anisotropy with increased Θ_IP_ in the LHPs NCs, resulting in higher η_out_ and η_ext_ of up to 32% and 25%, respectively.^[^ ^73^, ^98^ ^]^ Recently, controlling the layer numbers and the orientation in quasi‐2D perovskite films have been used to tailor the emission TDM orientation.^[^ ^98^, ^102^ ^]^ Specifically, Cui et al. used an in situ synthetic approach to obtain a face‐on orientation of monolayer NPLs, resulting in an 84±4% Θ_IP_ and reasonably high η_PL_ of 75% in the perovskite films (Figure 8d,e).^[^ ^98^ ^]^ The emission TDM orientation of in situ growth LHP NCs was characterized by both the angle‐dependent (AD) PL and the back focal plane (BFP) imaging techniques. They have also realized the highest η_out_ and η_ext_ of 31% and 23.3% (Figure 8f)^[^ ^98^ ^]^ respectively, in the green PeLEDs. The oriented LHP film morphology and rational η_PL_ are attributed to bulky organic ammonium cations, phenylbutylammonium bromide (PBABr), phenylethyl‐ammonium bromide (PEABr), and lithium bromide (LiBr) precursor solution. We will comprehensively discuss about fundamentals of LHP emission anisotropy in Section 5, including the LHP electronic structures, effects of NC geometry and anisotropic dielectric confinement, and self‐assembly and superlattices (see Sections 5.1 to 5.3).

### 3.6 Photon Recycling

Photon recycling refers to a process where photons are reabsorbed and re‐emitted multiple times within the same EML. This phenomenon has been observed in a number of semiconductor material systems having relatively small Stokes shift including LHP^[^ ^51^, ^103^, ^104^, ^105^ ^]^ and has been linked to improved device performance in both solar cells^[^ ^104^ ^]^ and LEDs.^[^ ^37^ ^]^ Indeed, since all the re‐emitting events are independent, the photon direction is randomized. If the photon was originally in a trapped mode, e.g. substrate or waveguide mode, this process can effectively act as a mechanism to recycle and extract photons that would otherwise be lost outside the escape cone. Although this process has been discussed in the literature, the role of TDM orientation on photon recycling efficiency has been largely overlooked. We first consider a simple system, where a flat emissive layer is placed on a glass substrate. The escape probability in a half‐space from an exciton of TDM angle θ relative to the substrate normal, *P*_out_, is given by^[^ ^106^, ^107^ ^]^

(2) $$P_{\mathrm{out}}=\frac{1}{2}-\frac{1}{2}\sqrt{1-{\left(\frac{n_{0}}{n_{1}}\right)}^{2}}\left(1+\frac{n_{0}^{2}}{2n_{1}^{2}}\left(1-\frac{3}{2}\sin^{2}\theta \right)\right)$$

where *n*_0_ and *n*_1_ are the refractive indices for the substrate and emissive layer, respectively. Note that Equation 2 considers a single emitter, so the ensemble escape probability would require angle averaging considering the TDM orientation distribution within the film, which is a function of the parameter Θ_IP_. For example, if the ensemble TDM is randomly oriented, Equation (2) is reduced to a well‐known relation considered in most literature as follows.

(3) $$P_{\mathrm{out}}^{\mathrm{isotropic}}=\frac{1}{2}\left(1-\sqrt{1-{\left(\frac{n_{0}}{n_{1}}\right)}^{2}}\right)$$

**Figure 9:**
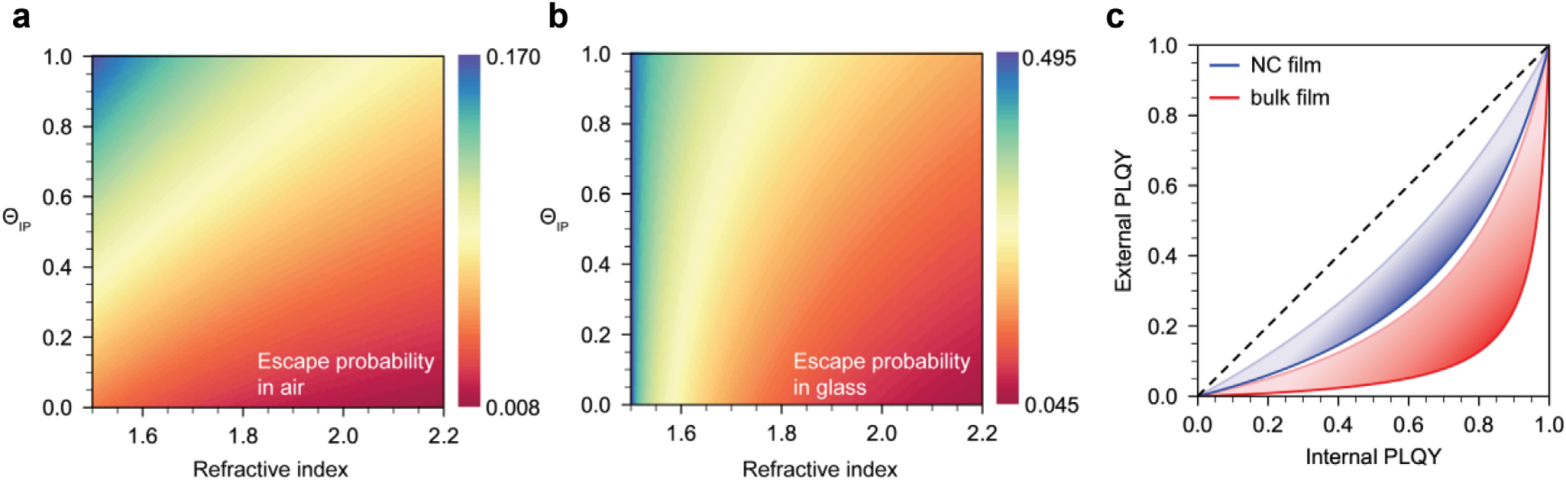
a,b) Calculated photon escape probability as a function of EML refractive index and TDM orientation Θ_IP_. c) Deviation of external η_PL_ from the internal η_PL_ resulting from photon recycling. The blue lines correspond to the parameters for a MA/FAPbBr_3_ NC thin film while the red lines to a bulk film of MAPbX_3_. The shaded area highlights the dependence on Θ_IP_, evolving from dark (Θ_IP_ = 0) to light (Θ_IP_ = 1) shaded areas.

Accordingly, **Figure** **9**a,b presents the calculated escape probabilities in air and glass as a function of EML refractive index and TDM orientation. Using the probability values, one can calculate the external η_PL_ measured in air, ϕ_ext_, given the parameters of internal η_PL_, ϕ, and the EML optical density, OD, at the emission wavelength following:^[^ ^37^, ^51^, ^108^ ^]^

(4) $$\phi_{\mathrm{ext}}=\frac{\phi \eta_{\mathrm{esc}}^{\mathrm{tot}}}{1-\left(1-\eta_{\mathrm{esc}}^{\mathrm{tot}}\right)\phi }$$

where $\eta_{\mathrm{esc}}^{\mathrm{tot}}$ is the total light escaping efficiency given by: $\eta_{\mathrm{esc}}^{\mathrm{tot}}=10^{-\mathrm{OD}/2}\left(P_{\mathrm{out}}^{\mathrm{air}}+P_{\mathrm{out}}^{\mathrm{glass}}+10^{-\mathrm{OD}}\left(P_{\mathrm{out}}^{\mathrm{glass}}-P_{\mathrm{out}}^{\mathrm{air}}\right)\right)$, and $P_{\mathrm{out}}^{\mathrm{air}}$ and $P_{\mathrm{out}}^{\mathrm{glass}}$ are functions of TDM orientation and refractive index of EML. Figure 9c displays the effect of photon recycling and TDM orientation in two model systems: a thin EML of MA/FAPbBr_3_ NCs^[^ ^73^ ^]^ and a thicker EML of polycrystalline bulk MAPbX_3_.^[^ ^51^ ^]^ Photon recycling can have a significant impact on device efficiency particularly for EMLs with intermediate η_PL_ values and high optical density. The effect of TDM orientation on the escape probability strongly affects the photon recycling efficiency especially for EMLs with high Θ_IP_, thus revealing a balance between photon recycling and outcoupling efficiency that limits their synergetic effect. Lately, the researchers have also observed that the device architecture of PeLEDs also plays a significant role on the photon recycling. For example, Greenham and co‐workers demonstrated a near infrared PeLED with a η_ext_ of 23.9% using a thin transparent conducting electrode, ITO, layer that strongly suppresses the parasitic absorption loss, thus enhancing the photon recycling fraction from 2.3% to 4.8%.^[^ ^109^ ^]^ Upon reducing the ITO electrode thickness from 150 nm to 15 nm, they observed ≈10% increase in the transmittance of ITO film that resulted only 1% absorption in the ITO layer. Through computational simulations, they have also demonstrated that the maximum η_ext_ of PeLEDs could be attained up to 36.6% by harnessing the photon recycling in 180 nm thick LHP layer. Considering the η_ext_ maxima, the suppression of parasitic absorption facilitates photon recycling in thick LHP layer that possesses a huge lead over microcavity effect in the thin LHP layer.

## 4 Synergetic Light Outcoupling Approaches

Motivated by the practical limitations and tradeoffs associated with the individual light extraction techniques, it becomes desirable to explore synergetic outcoupling techniques for efficient light extraction minimizing the substrate, waveguide, absorption, and SPP modes. Indeed, a large fraction of radiation power generated in the PeLEDs are dissipated in the lossy modes, which not only lead to poor η_out_ but also generate the Joule heating effect that considerably diminishes the device operational stability. However, because of the competitive nature between different modes,^[^ ^110^ ^]^ it remains challenging to systematically outcouple multiple modes at the same time and synergetic effects have to be carefully considered. To enhance the η_out_ of PeLEDs, complementary light out‐coupling mechanisms must be synchronized to avoid any competing losses. We invite the readers to visit a recent review article discussing specifically the role of two competing light‐outcoupling techniques, photon recycling, and microcavity, to overcome the ray‐optical limit of PeLED devices.^[^ ^110^ ^]^ Note that choosing between photon recycling and microcavity techniques for enhancing the *η*_out_ arises from their fundamental working principles. Taking the photon recycling mechanism as an example, the *η*_out_ is enhanced due to reabsorbtion and re‐emission of trapped photons from thick EMLs (>50 nm), capitalizing on effective radiative recombination. However, the increase in cavity length leads to a reduction in light extraction if the EML is utilized in a strong microcavity, complicating their simultaneous optimization. On the contrary, Benisty et al. have claimed the positive impact of a carefully designed microcavity on photon recycling.^[^ ^111^ ^]^ Therefore, a comprehensive experimental investigation of the interaction between these two strategies needs to be carried out to understand its role in determining device efficiency.

Very recently, there have been several attempts to explore synergetic approaches to maximize η_out_. For example, by combining the bioinspired moth‐eye nanostructures (MEN) with the hemispherical lens, Shen et al. demonstrated η_out_ enhancement by more than two‐folds by extracting both substrate and waveguide modes.^[^ ^83^ ^]^ The device η_ext_ and η_CE_ increased from 13.4 to 28.2% and 40.9 to 88.7 cd A^−1^, respectively. Bai et al. demonstrated a maximum η_out_ of 41.82% in green PeLED devices by combining multiple light extraction techniques, including the alignment of refractive indices between the LHP EML and adjacent layers and interface engineering for modulating the critical light extraction angle.^[^ ^32^ ^]^ As a result, the device η_ext_ reached up to 30.84%. In combination with the low‐refractive‐index EML/HTL/ETL and perfectly horizontal TDM orientation, it has been shown that the device η_out_ is as high as 54%.^[^ ^73^ ^]^ Accordingly, it is possible to overcome the 50% *η*_ext_ barrier without involving sophisticated fabrication or addition of layers, given the near‐unity *η*_int_ of colloidal LHP NCs.

## 5 Understanding Emission Anisotropy in LHP NCs

**Figure 10:**
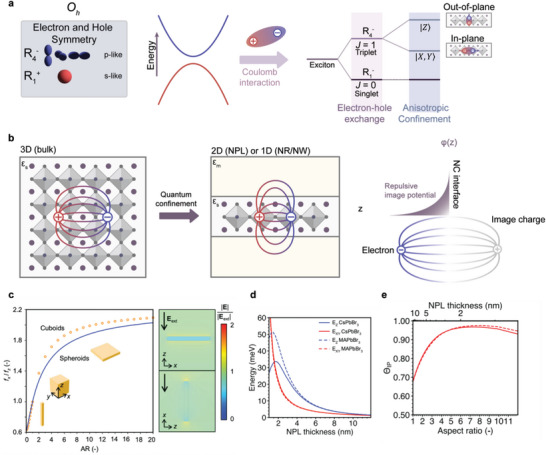
a) Schematic diagram visualizing the electronic structure of LHP NCs and the exciton fine structure. b) Schematic illustration for the effect of dielectric confinement in anisotropic nanostructures on the electron–hole Coulomb energy and the single carrier energy via the image potential. c) Ratio between the in‐plane and out‐of‐plane local field factors in anisotropic LHP NCs of different shapes. Right insets: comparison of the relative internal electric field within LHP NPLs when experiencing a vertical (top) and horizontal external electric field. Reproduced with permission.^[^ ^73^ ^]^ Copyright 2022 from Springer Nature. d) Fine splitting of the triplet manifold in CsPbBr_3_ and MAPbBr_3_ NPLs as a function of thickness. e) Theoretical TDM orientation parameter Θ_IP_ for a single CsPbBr_3_ and MAPbBr_3_ NPL as a function of thickness and aspect ratio. Reproduced with permission.^[^ ^100^ ^]^ Copyright 2022 from American Chemical Society.

Preferential TDM orientation in thin EMLs of LHP NCs results from the complex interplay of several factors, spanning multiple characteristic length scales.^[^ ^100^ ^]^ Here we bridge different levels of understanding (**Figure** **10**). At the quantum–mechanical level, the TDM orientation is largely dominated by the electronic structure of individual nanocrystals (Figure 10a,b). Next, the dielectric confinement of LHP NCs is determined by the dielectric contrast between the NCs and the surrounding capping ligands. Both these factors are strongly dependent on the NC shape and geometry and therefore become the critical design parameter to engineer the radiation pattern of individual NCs (Figure 10c). Lastly, at the ensemble level, the TDM orientation is fundamentally dependent on the orientational distribution of individual NCs within the EML film, thus involving ordering and crystallinity of superlattices and assemblies. Indeed, a notable advantage for the NC solid films, as compared to the solution‐processed bulk and quasi‐2D counterparts, is the possibility of decoupling the NC shape (controlled by colloidal synthesis) from NC assembly (controlled by film processing). In this section, we will provide in‐depth discussion about different levels of physical mechanism, which could offer a rationale to guide researchers in the optimization of PeLED outcoupling via TDM engineering, with specific emphasis on anisotropic NCs, including NPLs), nanorods (NRs), and nanowires (NWs), which have been extensively studied in the literature.^[^ ^112^, ^113^ ^]^

### 5.1 Electronic Structure

The LHP has a direct bandgap at the R point of the Brillouin zone, which is isomorphic to the Γ point, between a sixfold conduction band and a two‐fold valence band.^[^ ^11^, ^114^, ^115^ ^]^ These states can be classified according to the irreducible representations of the crystal point group (O_h_ for cubic LHP), which prescribe the possible spatial symmetry of Bloch functions that are compatible with transformations preserving the crystal structure. Accordingly, the valence band states have even parity that belongs to the scalar representation $R_{1}^{+}$, while the conduction band states have odd parity that belongs to the 3D vector representation $R_{4}^{-}$. This symmetry originates from the atomic orbital character of the Bloch states and is therefore customary to refer to the valence band states as *s*‐like (orbital angular momentum l = 0) and to the conduction band states as *p*‐like (l = 1). The additional degeneracy is simply the Kramers degeneracy.

As illustrated in Figure 10a, with the addition of spin–orbit interaction, the Bloch functions are eigenstates of the total angular momentum J and transform according to the double group representations: the valence states transform as a $R_{6}^{+}$(J = 1/2) state while the conduction states are split into the fourfold $R_{8}^{-}$ (J = 3/2) and twofold $R_{6}^{-}$ (J = 1/2) states. The emission properties of NCs are determined by excitons, which are electron/hole pairs correlated via their Coulombic interaction.^[^ ^116^, ^117^, ^118^ ^]^ As a consequence, the lowest‐energy transitions are associated with the recombination of a fourfold exciton manifold generated by conduction–valence pair states between the $R_{6}^{-}$ and $R_{6}^{+}$ bands $\left(R_{6}^{-}⊗R_{6}^{+}\right)$.

The total exciton wavefunction is then composed of these Bloch states, which only depend on the crystal structure and control the selection rules and the polarization of the allowed optical transitions, and of an envelope function, which encodes the effects of quantum confinement and depends on the NC shape and size. Specifically, depending on the ratio of NC size (*L*) and bulk exciton Bohr radius (*a_X_*), three regimes of quantum confinement can be defined. In the weak confinement regime (*L* > *a_X_*), the exciton center‐of‐mass translational motion is confined like an atom in a box and the electron and holes are strongly correlated. In the opposite case of strong confinement (*L* < *a_X_*), the NC properties are dominated by the electron and hole quantum size levels. The transition region is known as the intermediate confinement. LHP NCs are typically in weak confinement (≥10 nm) due to their extremely fast ionic coprecipitation kinetics, which makes the control of nucleation for the synthesis of small NCs challenging. We noticed that recently a novel synthetic route for the controlled synthesis of spheroidal LHP NCs with small size and strong excitonic effects has been reported.^[^ ^119^ ^]^

On the other hand, the anisotropic NCs like NPLs, NWs, and NRs, are characterized by one or more dimensions strongly confined. The degeneracy of the fourfold exciton manifold is further lifted by the electron–hole exchange interaction into a dark singlet ($R_{1}^{-}$) and a threefold bright triplet ($R_{4}^{-}$) state. It is noteworthy here to clarify the usage of the terms “singlet” and “triplet” to avoid confusion. Conventionally, the terms are used to indicate the symmetric and antisymmetric combinations of two‐electron spin eigenstates that diagonalize the exchange interaction in a two‐particle system like the He atom or a Frenkel exciton in organic semiconductors. In this latter case, it has a particular technological significance in the field of OLEDs. Since the light–matter interaction does not affect the spin part of the states involved in an optical transition, spin singlets are always bright and spin triplets are always dark as their recombination would require a spin flip. This conclusion initially seems at odds with most of the LHP literature where the singlet state is considered as the dark state while the triplet states are optically active. Indeed, in this context, the terms “singlet” and “triplet” are simply used in relation to the degeneracy of the states. In addition, the conduction states are not simple eigenstates of the spin operator but spin‐orbit states that involve superpositions of up and down spin states, further complicating the analysis. For example, an analysis of the spin content of the “singlet” J = 0 exciton reveals that it has pure spin triplet character and therefore is optically dark,^[^ ^114^ ^]^ thus reconciling the nomenclature.

PL and EL in NCs involve thermal relaxation into the lowest‐lying exciton states, so both processes are therefore controlled by their fine structure,^[^ ^120^ ^]^ explaining its importance to understand the TDM orientation in NCs. A combination of low dielectric constant and small exciton Bohr radius makes electron–hole exchange interaction particularly significant in LHP materials, even at room temperature. The electron–hole exchange can be split into two contributions: the short‐ and long‐range exchanges.^[^ ^120^, ^121^ ^]^ The short‐range exchange, *H* ^SR^ has the form of a contact spin–spin interaction:^[^ ^122^ ^]^

(5) $$H^{\mathrm{SR}}=\frac{1}{2}C_{\mathrm{ex}}\Theta \left(\hat{I}-\sigma_{h}\cdot \sigma_{e}\right)$$

where *C*_ex_ is the short‐range exchange constant of the material, Θ is the electron–hole overlap which depends on the exciton envelope function, $\hat{I}$ is the 4 × 4 identity matrix, and σ_*e*,*h*_ the Pauli spin matrices for the electron and hole.

The long‐range exchange contribution, *H* ^LR^, can be understood as the Coulomb dipole–dipole interaction associated with the exciton transition polarization^[^ ^123^, ^124^, ^125^ ^]^

(6) $$H_{ij}^{\mathrm{LR}}=\int dV\int dV^{\prime }\left(-\nabla \cdot P_{i}\left(r\right)\right)\frac{1}{\varepsilon \left|r-r^{\prime }\right|}\left(-\nabla \cdot P_{j}\left(r^{\prime }\right)\right)$$

where *P_i_* (*r*) is the exciton polarization associated with charge–density ρ(*r*) = −∇ · *P*_**i**_(*r*) as a function of position vector *r*. The longitudinal exciton states, whose polarization direction is parallel to the exciton propagation direction *K*, are unaffected (∇ · *P_i_* (*r*)  =  0), and therefore the long‐range exchange interaction is responsible for the longitudinal‐transverse splitting of exciton states in the bulk.^[^ ^126^ ^]^

Consider an isolated cubic NC, because the triplet states |*X*〉,  |*Y*〉,  |*Z*〉 are degenerate, leading to an effectively isotropic electronic structure, the ensemble TDM is randomly oriented. However, the exchange interaction is strongly dependent on the exciton envelope, so the triplet degeneracy can be lifted in anisotropic NCs like NPLs, NRs, and NWs.^[^ ^127^, ^128^ ^]^ In particular, in LHP NPLs, bright triplet fine splittings of up to 20 meV have been theoretically predicted^[^ ^127^, ^129^ ^]^ and experimentally^[^ ^101^ ^]^ observed, leading to an increase in the population of the in‐plane |*X*, *Y*〉 states even at room temperature. In addition, anisotropic confinement leads to a modification of the conduction Bloch states that changes the mixing between the *p*‐like states depending on the confinement direction. The combination of these two factors contributes to the strongly anisotropic PL emission, particularly in NPLs. Notably, although lowering the crystal symmetry to tetragonal or orthorhombic also leads to fine splitting within the bright triplet manifold,^[^ ^11^, ^115^, ^120^, ^130^ ^]^ the magnitude is rather negligible compared with the effect of strong quantum confinement.

### 5.2 The effect of Shape and Anisotropic Dielectric Confinement

The major contributor to the TDM anisotropy in individual LHP NCs is the dielectric contrast between NCs and the surrounding medium, which is composed of capping ligands. On one hand, the relatively lower dielectric constant of the ligand shell leads to a repulsive potential, caused by the “image charges,”^[^ ^131^ ^]^ which push electrons away from the NC surface causing an increase in the energy of the carriers’ quantum size levels.^[^ ^116^ ^]^ On the other hand, in strongly confined NCs, the reduction of the dielectric screening due to the penetration of the electric field of the carriers in the surrounding medium, strongly enhances the electron–hole Coulomb interaction, thereby resulting in a modification of the exciton envelope function and the long‐range exchange fine splitting.

Among all mechanisms inducing TDM orientation in perovskite NCs, the most significant contribution is the renormalization of the local electric field as a consequence of dielectric confinement,^[^ ^132^, ^133^, ^134^, ^135^, ^136^, ^137^ ^]^ following $E_{i}^{loc}=f_{i}\left(\tilde{\in },\zeta \right)E_{i}^{ext}$, where *f_i_* are the local field factors, function of the dielectric contrast $\tilde{\in }=\in^{in}/\in^{out}$ and the NC shape through the geometric parameters ζ. This renormalization can depend strongly on direction like in the case of anisotropic NC. Figure 10c shows the local field factors for APbBr_3_ NCs as a function of their aspect ratio. A high value of aspect ratio corresponds to NPLs; NRs are associatedA with NCs of aspect ratios lower than unity. The internal field is strongly anisotropic, with high screening along the quantum‐confined dimensions, whereas in the extended dimensions, the field factors remain unchanged.

Since the internal field in the NC mediates the light–matter interaction, it strongly alters the probability of exciton recombination which is a function of the exciton polarization. Taking LHP NPLs as an example, the dielectric anisotropy leads to a reduction of the out‐of‐plane exciton emission by a factor |*f_i_* |^2^, thereby yielding a preferentially in‐plane TDM distribution even for isotropic electronic structures.^[^ ^73^ ^]^ The anisotropic emission has been observed even for ensembles of weakly confined anisotropic MA/FAPbBr_3_ NCs leading to an increased outcoupling efficiency in PeLEDs.

**Figure 11:**
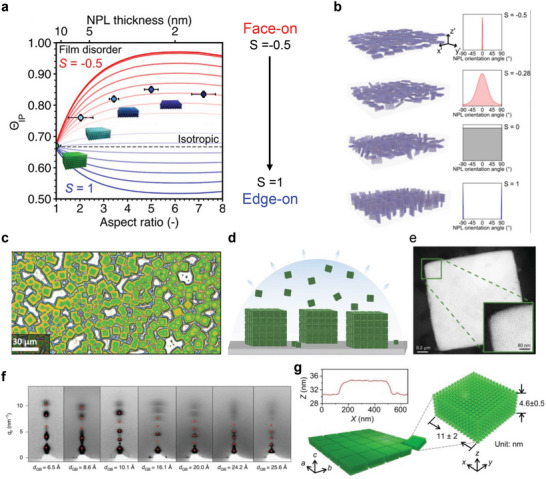
a) Effect of assembly disorder on the TDM orientation Θ_IP_ in LHP NPLs. b) Schematic diagram illustrating different uniaxial orientational distributions in anisotropic LHP NC films. Reproduced with permission.^[^ ^100^ ^]^ Copyright 2022 from American Chemical Society. c) Optical micrograph of LHP NC superlattices. Reproduced with permission.^[^ ^186^ ^]^ Copyright 2021 from American Chemical Society. d) Schematic illustration for the self‐assembly of cubic LHP NCs forming superlattices through solvent evaporation. e) Scanning electron microscope image of a simple cubic CsPbBr_3_ superlattice with the individual NCs still visible. Reproduced with permission.^[^ ^145^ ^]^ Copyright 2018 from Springer Nature. f) GIXD crystallographs for LHP NPL thin films showing lamellar superlattices of face‐on orientation. The individual lamellar peaks clustered along *q_z_* axis informs the interlayer thickness, allowing the estimation of capping ligand thickness. Reproduced with permission.^[^ ^20^ ^]^ Copyright 2020 from Springer Nature. g) Schematic illustration for the face‐on ordered assembly of LHP anisotropic NC superlattices and atomic force microscope height profile of an extended superlattice in a spin‐coated film. Reproduced with permission.^[^ ^73^ ^]^ Copyright 2022 from Springer Nature.

On the other hand, in the case of strongly quantum‐confined NPLs, the effect of dielectric anisotropy has to be considered together with the modification of the conduction Bloch states upon quantum confinement, involving the anisotropic fine splitting discussed earlier. Taking all effects into account, **Figure** **11**a presents the calculated TDM orientation of LHP NPLs as a function of their thickness. A maximum of Θ_IP_ = 0.91 in thin NPLs with a thickness of 3 monolayers. Interestingly, for ultrathin NPLs, there exists a saturation of Θ_IP_ as a consequence of the energy inversion of the out‐of‐plane and in‐plane exciton states.

### 5.3 The Role of the Ensemble Disorder: Self‐Assembly and Superlattices

In the previous sections, the discussion has been focused on the physical origin of emission anisotropy at the level of single NCs. However, regarding the actual implementation of PeLEDs, the TDM orientation represents a collective property of NC ensembles and it strongly depends on how individual NCs assemble to form thin films.^[^ ^100^ ^]^ Figure 11b presents the effect of the orientational distribution of NPLs on the TDM orientation parameter Θ_IP_. Indeed, the emission angular characteristics of NC thin films depend on the relative orientation between the ensemble TDM of exciton states in individual NCs, which is directly related to the spatial orientation of individual NCs and their long‐range packing order. The face‐on assembly of NPLs can in‐principle maximize Θ_IP_ and *η*_out_, while a degree of randomization would compromise the in‐plane TDM orientation, even using strongly confined NPLs with highly anisotropic emission. On the other hand, the TDM distribution in the edge‐on assemblies, despite a considerably lowered outcoupling efficiency, could generate linearly polarized light, and PeLEDs with a degree of polarization up to 74.4% have been recently demonstrated.^[^ ^101^ ^]^

Similar approaches can be also exploited for 1D structures like NWs and NRs. However, the realization of high‐degree linear polarization demands the fabrication of textured films of control over the azimuthal orientation of NWs/NRs, which remains very challenging. A key advantage of colloidal NCs, as compared to the bulk/quasi‐2D LHP films, is the tendency for self‐assembly into ordered superstructures known as superlattices.^[^ ^138^, ^139^, ^140^ ^]^ Upon aggregation, the NCs undergo a phase transition accompanied by the nucleation and growth of highly ordered superlattice domains, which is a process underlying the subtle balance between the attraction forces between NC cores and the elastic repulsion between the ligand shells.^[^ ^141^ ^]^ In recent years, superlattices of LHP NCs have been extensively studied,^[^ ^142^, ^143^, ^144^ ^]^ in particular following the experimental observation of superfluorescence in 3D superlattices of CsPbBr_3_ nanocubes with simple cubic packing (Figures 11c–e).^[^ ^145^, ^146^ ^]^

Superlattices are commonly produced either by controlled solvent evaporation or destabilization of the colloidal solution.^[^ ^138^ ^]^ The former approach is the most common and ordered NC arrays can be simply formed at the air–liquid interface upon evaporation of a droplet of colloidal solution. The same procedure is often carried out in a tilted vial to obtain control over the meniscus angle and direction during solvent drying. The immiscibility of certain polar solvents like diethylene glycol with the nonpolar solvents used to disperse the NCs, has been exploited to grow highly ordered superlattice films at the liquid–liquid interface which can be transferred to a substrate by draining the polar liquid in a Teflon trough.^[^ ^47^, ^147^ ^]^ This technique has been often used with colloidal NCs of II‐VI semiconductors. For example, Gao et al. showed that the NPL orientational distribution could be tuned from edge‐on to face‐on with the addition of oleic acid to the polar subphase.^[^ ^47^ ^]^ However, the poor stability for LHP NCs in most polar solvents considerably limits the feasibility in practice. Baranov et al. showed that the fluorinated solvent perfluorodecalin can be used to grow CsPbBr_3_ superlattices at the liquid–liquid interface, through the colloidal destabilization and solvent diffusion into the nonpolar colloidal solution.^[^ ^148^ ^]^ Notably, the anisotropic LHP NCs such as NPLs tend to form lamellar superlattices due to the strong face‐to‐face NC interaction.^[^ ^149^ ^]^ NPLs tend to form ordered assemblies even under fast solvent evaporation induced during spin‐coating.^[^ ^101^, ^150^ ^]^ The transition between the edge‐on and face‐on orientations can be triggered by controlling the vapor pressure of the nonpolar solvent, although the opposite behavior had been observed in spin‐coated and drop‐casted samples. Indeed, more in‐depth investigations are required to decipher the kinetics of NPL self‐assembly.

Note that despite the high‐quality superlattices produced by solvent evaporation, practical application to PeLEDs demands the development of self‐assembly protocols that are scalable to large‐area devices and compatible with PeLED fabrication and underlying carrier transport layers. In particular, self‐assembly strategies should be tested directly within the PeLED device stacks as the surface energy of the substrate has been shown to have a strong influence on the orientational distribution of LHP NCs.^[^ ^73^ ^]^ The PL emission anisotropy and angular dependence are highly sensitive to the packing order of NCs. In order to fully decouple the TDM orientation of individual NCs and the morphological characteristics of their assemblies, detailed grazing incidence X‐ray diffraction (GIXD) characterization, potentially in situ, is highly recommended (examples see Figure 11f,g). We anticipate that the incorporation of highly ordered superlattices as EMLs in devices is an important research direction for next‐generation PeLEDs, not only because of the increased outcoupling efficiency via the TDM orientation engineering but also taking advantage of the improvement in charge transport offered by ordered NC assemblies, as a consequence of bandlike transport through delocalization of the exciton wavefunction to neighboring NCs.^[^ ^151^, ^152^, ^153^, ^154^, ^155^, ^156^, ^157^, ^158^, ^159^ ^]^ Recently, epitaxial LHP superlattices have emerged as an attractive alternative for conventional bulk (poly‐ or single‐crystalline) active layers in LHP solar cells, in addition to superlattices created by the self‐assembly of LHP colloidal NCs.^[^ ^160^ ^]^ These highly ordered superstructures are typically grown from precursor solutions at the solid–liquid interface on an underlying LHP crystal template. The absence of grain boundaries together with the perfect crystalline ordering of templated growth, gives rise to improved charge carrier diffusion length and dynamics in solar cells.^[^ ^160^ ^]^ Although the low exciton binding energy of bulk LHP may hinder the application of epitaxial LHP superlattices in PeLEDs, Zhang et al. have recently managed to prepare twisted bilayer LHP superlattices where the Moiré confining potential, controlled by the twisting angle, configures itself as a promising extrinsic strategy to increase the exciton binding energy in bulk LHP.^[^ ^161^ ^]^

## 6 Characterization Techniques for in‐Plane Emission Directionality

In the past two decades, numerous techniques have been proposed to characterize the emission anisotropy of EMLs made by organic, inorganic QDs, and LHP emitters. Here we specifically emphasize on the following techniques, including variable angle spectroscopic ellipsometry (VASE), angle‐dependent photoluminescence (ADPL) spectroscopy, Fourier or back focal plane (BFP) imaging, and GIXD, which provide fundamental insights into the TDM by analyzing emission intensity and wavelength.^[^ ^99^, ^162^, ^163^, ^164^, ^165^, ^166^, ^167^, ^168^ ^]^ Note that other specialized techniques, such as single‐emitter spectroscopy, single‐molecule microscopy, impedance spectroscopy, Kelvin probe measurement, and displacement current measurement, can also be used to determine the TDM orientation of light‐emitting materials,^[^ ^169^, ^170^, ^171^, ^172^, ^173^, ^174^ ^]^ we encourage the readers to explore the relevant content if interested.

### 6.1 Variable Angle Spectroscopic Ellipsometry (VASE)

**Figure 12:**
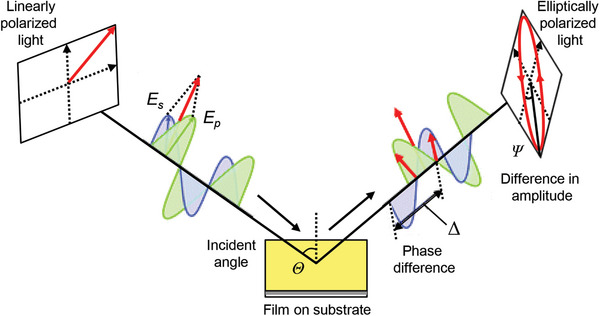
Schematic illustration of the VASE analysis. Reproduced with permission.^[^ ^163^ ^]^ Copyright 2009 from Elsevier.

**Figure 13:**
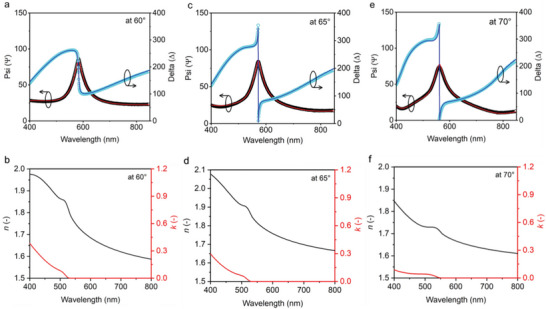
A set of measured data using the VASE analysis. Spectroscopic ellipsometry Ψ and Δ experimental data and corresponding Tauc–Lorentz fittings for anisotropic NCs superlattice FA_0.5_MA_0.5_PbBr_3_ films *n* and κ values at a,b) 60°, c,d) 65°, e,f) and 70°. Reproduced with permission.^[^ ^73^ ^]^ Copyright 2022 from Springer Nature.

VASE is one of the most common methods to investigate the optical anisotropy of EML thin films. Specifically, a beam of linearly polarized white light is incident on the film surface with an incident angle close to the Brewster angle. After specular reflection, light is elliptically polarized, yielding the complex reflectance ratio between the reflection coefficients of *s*‐ and *p*‐polarized (*s*‐ and *p*‐pol) light. One could then measure the amplitude Ψ and phase shift Δ, which are fitted with appropriate optical models, such as Sellmeier, Cauchy, and Tauc–Lorentz, to obtain the refractive index (*n*), extinction coefficient (κ), and film thickness, as illustrated in **Figure** **12**. Note that the extracted parameters are very sensitive to the thickness and refractive index of the EML thin film. **Figure** **13** presents a set of measured phase and amplitude data (top panels) of mixed cation FA_0.5_MA_0.5_PbBr_3_ anisotropic NC film at three different incident angles, 60^⁰^, 65^⁰^, and 70^⁰^ and their respective *n* and κ values (bottom panels), extracted by fitting the Tauc–Lorentz model.

The extinction coefficient κ, which is the imaginary part of the complex refractive index, is given by κ  =  (λ/4π)α, where λ is the emission wavelength and α is the absorption coefficient of the film. Accordingly, the extinction coefficient is proportional to the absorption at a given angle, and thus has a larger value in the ensemble TDM direction of EML. In 2004, Lin et al. reported the first observation of uniaxial anisotropy in thermally evaporated EMLs of fluorescent organic emitters.^[^ ^162^ ^]^ Subsequently, Yokoyama meticulously used this technique to investigate the molecular orientation of organic light‐emitting materials taking into account the shape anisotropy of organic molecules.^[^ ^163^ ^]^

The same principle can be readily applied to LHP EMLs. Considering an EML thin film having a collection of TDMs, the ensemble TDM orientation should be reflected in the anisotropy of the extinction coefficients. In other words, κ should have different values in different directions (incident angles). If the TDM vector is randomly oriented within the EML in parallel to the substrate plane, the distribution is uniaxial and can be informed by only two quantities: the ordinary and extraordinary extinction coefficients. When there exists a large difference between the two parameters, it implies a strongly anisotropic film, or preferentially oriented TDM. Specifically, one can estimate the TDM orientation in a given EML film using the orientation parameter *S* following:

(7) $$S=\frac{\kappa_{e}^{\max}-\kappa_{o}^{\max}}{\kappa_{e}^{\max}-2\kappa_{o}^{\max}}=\frac{1}{2}\left(2-3\Theta_{\mathrm{IP}}\right)$$

where κ_*e*_ and κ_*o*_ are the extraordinary and ordinary extinction coefficients, respectively.

### 6.2 Angle‐Dependent Photoluminescence (ADPL) Spectroscopy

**Figure 14:**
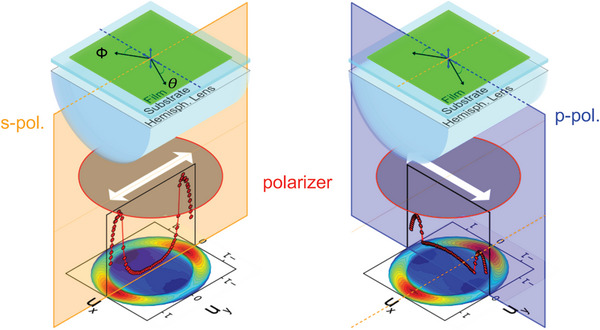
Schematic illustration for the ADPL setup that determines the ensemble TDM orientation in a LHP EML thin film coated on glass. Reproduced with permission.^[^ ^100^ ^]^ Copyright 2022 from the American Chemical Society.

The ADPL spectroscopy is one of the most popular techniques extensively used to characterize the PL emission anisotropy, including the determination of TDM orientation in EML thin films made by organic molecules, chalcogenides core–shell quantum dots (QDs), and LHP NCs. As shown in **Figure** **14**, this technique consists of a charge‐coupled device (CCD) spectrometer and polarizer with a large hemicylindrical prism, which allows the extraction of photons with a normalized wave vector 1 < *k*/*k*_0_ < *n*_glass_ that usually dissipates in the substrate modes. The experimental implementation of ADPL for the determination of TDM orientation was initially proposed by Frischeisen et al.^[^ ^164^ ^]^ The measurement usually contains two steps as follows. First, the EML thin film is coated on a glass substrate, followed by mounting the dielectric stack on top of the hemicylindrical prism with a refractive index matching fluid, to ensure no air gap at the substrate‐lens interface. Next, the ADPL measurement is carried out by attaching an excitation light source on the substrate to excite the thin‐film sample. Both the excitation source and detector are either vertically or horizontally aligned to properly align the excitation and detection pathways.

In practice, note that the full light beam from the excitation spot should be collected into the detector to collect all emitted photons. Insufficient light collection could lead to inaccurate measurement that yields incorrect TDM orientation. In addition, one should use an appropriate excitation source to ensure sufficient absorption without undesirable interference. Recently, Hänisch et al. developed a refined ADPL system to address the issue of light collection upon excitation by incorporating additional lenses between the hemicylindrical prism and detector.^[^ ^175^ ^]^ The focal point of these lenses coincides with the hemicylindrical prism and detector to redirect the maximum out‐of‐focus light from the thin film sample to the detector. The improved ADPL technique results in a signal‐to‐noise ratio approximately one order of magnitude higher than that of traditional ADPL systems. In addition to chromatic aberration, the spherical aberration of the hemicylindrical prism could also influence the experimental results. Indeed, the ADPL is a typical angular PL intensity measurement involving simultaneous sweeping of both polarizations (θ) and viewing angles (ϕ). Compared to the *k*‐space imaging technique, the ADPL technique allows forward and backward scans of viewing angle within $\pm 90^{\circ }$ with a variable step size of ≥ 0.1°. Likewise, the polarization angle can also be varied between 0° and 90°, where θ  =  0° corresponds to *p*‐ and θ  =  90° to *s*‐polarization.

Finally, one can fit the *s*‐ and *p*‐polarized PL intensities as a function of viewing angle using optical simulations, with the ensemble TDM orientation, Θ_IP_, as the only fitting parameter. Note that in order to properly fit the experimental data, it is vital to use accurate film thickness and *n*, κ values for EML materials characterized by spectroscopic ellipsometry. Furno et al. proposed a hypothetical theory to inform the angular radiation pattern of EMLs using the classical electromagnetic model.^[^ ^176^ ^]^ An arbitrarily oriented dipole can be described as the superposition of in‐plane and out‐of‐plane dipoles emitting transverse magnetic (TM) and transverse‐electric (TE) polarized light, respectively. In short, the ADPL represents the most popular technique for characterizing the emission anisotropy and ensemble TDM orientation because it can resolve emission into the substrate mode with a rather simple instrumentation.

**Figure 15:**
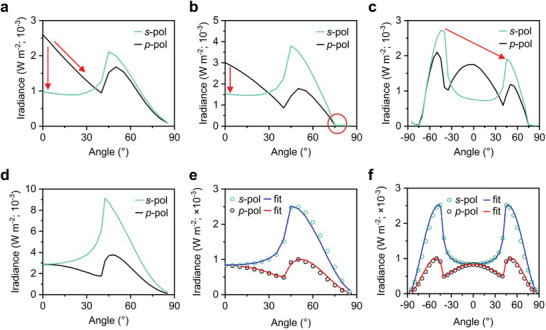
The ADPL profiles for LHP films illustrating the influence of photobleaching effects on the experimentally characterized *s*‐polarized and *p*‐polarized PL intensities as a function of viewing angle (a, b, and c), in comparison with the successful measurements and corresponding theoretical fittings (d, e, and f).

It is noteworthy that, as compared to the *k*‐space/back focal plane (BFP) imaging, there are a number of precautions to properly carry out the measurement, including the calibration of a polarizer and stage angle, appropriate substrate thickness, and optimization of excitation light energy to mitigate the photobleaching issue of EMLs. In particular, the quantum efficiency and photostability of the EML represent the most important considerations. The ADPL is not a reliable method to correctly characterize the TDM orientation of highly photosensitive emitters with low η_PL_, because the entire measurement could take several minutes, resulting in EML degradation. The low η_PL_ emitters demand high excitation power and long acquisition time to reach high signal‐to‐noise ratios, which further degrade the material. The photobleaching effect can result in a misalignment between the *s*‐ and *p*‐polarized emission profiles and intensities. **Figure** **15** presents a number of inconsistent profiles that one could observe in the measurement of photo‐bleached samples. Specifically, photobleaching of EMLs could lead to inconsistent *s*‐ and *p*‐polarized emission intensities at the front angle ($\phi =0^{\circ }$; Figure 15a,b). For a standard forward scan measurement from $\phi =0^{\circ }$ to $90^{\circ }$, it results in an overestimation of light being coupled to air within the critical angle (Figure 15a). Consider a forward scan measurement from $\phi =-90^{\circ }$ to $90^{\circ }$, it could cause an asymmetric ADPL profile (Figure 15c).

Accordingly, it is essential to record either both *s*‐ and *p*‐polarized emission intensities for measurements between $0^{\circ }$ to $90^{\circ }$ or $-90^{\circ }$ to $90^{\circ }$ (Figure 15d–f). The asymmetry in the ADPL profiles could be minimized by optimizing the pumping fluence of the excitation source. Besides, a small degree of misalignment in the zero position of the hemispherical lens and polarizer angle could also lead to inconsistent measurement, thus resulting in an incorrect value of TDM orientation. Finally, the ADPL measurement is also sensitive to the EML and substrate thicknesses, which could affect the signal‐to‐noise ratio because of photon loss from the substrate edges. Based on our experience, the most accurate measurement involves thin EML films of 30 ± 5 nm deposited on sub‐millimeter glass substrates, such as the coverslips.

### 6.3 Fourier or Back Focal Plane Imaging

An alternative technique to characterize the radiation pattern of NC thin films and therefore their TDM orientation is Fourier or the back focal plane (BFP) imaging.^[^ ^167^, ^168^, ^177^, ^178^ ^]^ This technique is based on the intrinsic property of converging lenses to perform 2D spatial Fourier transforms of the radiation fields. The radiation source, in this case, an EML thin film, is placed in the front focal plane of a high numerical aperture (N.A.) objective, which collects the emitted photons. On the BFP of the objective, an intensity map of the reciprocal space of the sample, *i.e*., the *k*‐space, is obtained, corresponding to the decomposition of the collected emission in its plane wave components. Therefore, in the BFP, each point corresponds to radiation emitted along the direction specified by the pair of in‐plane wave vectors $\left(\frac{k_{x}}{k_{0}},\frac{k_{y}}{k_{0}}\right)=\left(\sin\theta \cos\phi ,\sin\theta \sin\phi \right)$, where *k*_0_ is the wave vector in air.

**Figure 16:**
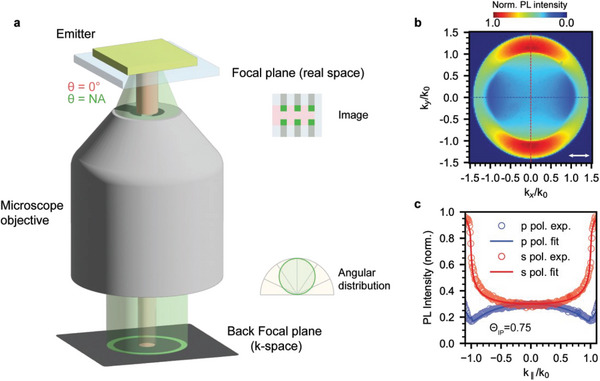
a) Schematic diagram for Fourier BFP imaging. The normal (red) and angular (green) photons are directed to different positions at the back focal plane, which therefore informs the angular information. b) An example of the BFP image generated from the PL emission of a thin film CsPbI_3_ NPL thin film deposited on glass. The annulus beyond $\frac{k}{k_{0}}>1$ of strong PL intensity corresponds to the radiation extracted from substrate modes captured by the immersion high‐NA objective. The dashed lines correspond to the *p*‐ and *s*‐ pol. cuts. c) Corresponding *p*‐pol. and *s*‐pol. profiles with the fitting curves used to estimate Θ_IP_. Reprinted with permission.^[^ ^97^ ^]^ Copyright 2024, Wiley‐VCH.

In practice, the objective's BFP hides within the objective body, such that a number of relay lens configurations are required to extract to an external image sensor.^[^ ^179^ ^]^ BFP imaging is the ideal characterization technique for samples that are prone to photodegradation as it provides complete mapping of angular characteristics generated from a radiation source, in a single measurement step without scanning in the angular and polarization space. The measurement is significantly fast, and even the sample experienced a degree of degradation during the measurement, in principle it does not affect the extracted TDM orientation Θ_IP_ value. The characterization of the TDM orientation of an EML film using the basic BFP imaging involves the following steps: i) the PL emission is collected with an oil immersion objective to allow for the extraction of the substrate modes, $1<\frac{k}{k_{0}}<N.A.$, and ii) a linear polarizer is placed before the imaging sensor to break the azimuthal symmetry of the radiation pattern for the extraction of *p*‐polarized profile, *I_p_* (θ), which is a linecut of the BFP image in the direction of the polarizer. The *p*‐polarized angular intensity is then fitted as prescribed in Section 6.2 to obtain the TDM orientation parameter Θ_IP_. For details please see **Figure** **16**.

Using an objective lens with a large numerical aperture, the BFP approach can characterize the TDM orientation across a wide range of viewing angles. The entire measurement typically takes on the order of milliseconds to seconds. It is important to note that choosing an objective lens with a larger aperture ratio is critical for capturing a wide angular range of BFP images, so that the substrate‐mode radiation can be collected. However, as the aperture ratio of the objective lens increases, image aberration increases. As a result, having an objective lens with an appropriate aperture angle is critical for acquiring an accurate BFP image while minimizing spherical aberrations. To select a suitable objective lens, one can use the equation *d_S_*  =  2*C_S_* α^3^, where *d_S_* is the spherical aberration contribution and *C_S_* is the spherical aberration at an angle α, highlighting the relationship between the aperture angle and the disk size with the least spherical aberration.

### 6.4 Grazing Incidence Wide‐Angle X‐Ray Scattering (GIWAXS) and X‐Ray Scattering

In earlier sections, we discussed the importance of the orientational distribution of NCs in determining the emission anisotropy and ensemble TDM orientation in EML thin films. The most direct quantification of the orientational distribution of NCs is the analysis of pole figures extracted from X‐ray diffraction on an area detector.^[^ ^180^, ^181^, ^182^ ^]^ The X‐ray characterization on thin films is usually performed with a high‐intensity monochromatic beam from a synchrotron radiation source at grazing incidence. The grazing incidence wide‐angle X‐ray scattering (GIWAXS) yields a 2D map of the scattered X‐ray radiation as a function of the scattering wave vector *q*  =  (*q_x_*, *q_y_*, *q_z_*), where *q_z_* and *q*_*x*,*y*_ are the out‐of‐plane and in‐plane components, respectively.^[^ ^182^, ^183^, ^184^ ^]^

For quantitative estimation, the raw data, after being converted to reciprocal space, is corrected for the curvature of the Ewald sphere. Indeed, in GIWAXS, due to large scattering angles and sample‐detector distance, the *q_z_* axis is perfectly probed and a small *q_y_* component has to be accounted for. This is usually solved by plotting the intensity in the *q_z_* − *q_r_* plane, where $q_{r}=\sqrt{q_{x}^{2}+q_{y}^{2}}$, leading to the so‐called “missing wedge.” It is important to note that this correction implies a fiber texture for the sample, i.e., a film with isotropic in‐plane orientation, and films with preferential in‐plane orientation should be analyzed with care. A NC film with no preferred orientation will result in Debye–Scherrer rings of uniform intensity. The diffraction pattern of oriented assemblies of NCs is instead characterized by intensity spots or arcs depending on the width of the orientational distribution. A major advantage for GIWAXS is its accessibility to the specific crystallographic orientations with respect to the substrate plane. The construction of a complete pole figure can allow the complete quantitative analysis of the film texture. It is particularly relevant for anisotropic NCs like NPLs, where the fraction of NPLs in face‐on or edge‐on configuration can be determined.

One can reconstruct a pole figure by selecting a specific Bragg peak in the GIWAXS image that extracts the azimuthal profile *I*(χ), integrated across the whole width of the peak. The features in the pole figure reflect populations of NCs with specific orientations. Particularly, the peaks centered at χ  =  0° or χ  =  90° correspond to the face‐on or edge‐on NC populations, respectively. The pole figure is usually corrected to account for the reduction of intensity with increasing χ by multiplying by a factor that is equal to sin χ in the case of fiber texture.^[^ ^182^ ^]^ The analysis of the orientational distribution of NWs or NRs is rather complicated owing to the possibility of in‐plane preferential orientation for the NCs in the substrate azimuthal direction. This is usually quantified by performing a ϕ‐scan,^[^ ^182^, ^185^ ^]^ where a diffraction peak is selected and its intensity is recorded as the sample is rotated along its out‐of‐plane *z*‐axis. A fiber‐textured film, as it is typically the case for NPL films in face‐on configuration, would lead to rings of uniform intensity along the azimuthal direction. Arcs or spots are instead a signature of preferred in‐plane orientations. This analysis is especially important for NW/NR or NPL films used to generate linearly polarized light as preferential in‐plane orientation is a prerequisite to obtain a high degree of polarization and it should be appropriately quantified as described.

**Figure 17:**
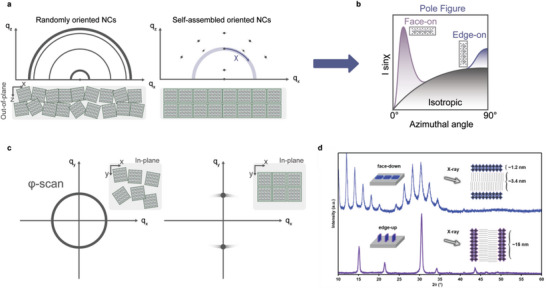
a) Schematic illustration for GIWAXS images of disordered (left) and ordered (right) assemblies of NCs. b) Schematic example of a pole figure extracted from an azimuthal cut along a Bragg peak in a GIWAXS image. c) Schematic illustration showing an example of a ϕ‐scan measurement for the quantification of the in‐plane texture of a NC film. d) Multilayer diffraction patterns for the face‐on and edge‐on LHP NPL assemblies. In the case of face‐on NPLs multiple diffraction fringes of the Bragg peaks emerge. Reproduced with permission.^[^ ^150^ ^]^ Copyright 2023 from the American Chemical Society.

We conclude this section by discussing multilayer diffraction using the powerful X‐ray diffraction technique that has recently been introduced for the characterization of LHP NC superlattices (**Figure** **17**d).^[^ ^146^, ^186^ ^]^ The measurement is performed in the Bragg–Brentano geometry on a conventional tabletop X‐ray diffractometer by performing a θ: 2θ scan. This ensures that the scattering vector *q* is perpendicular to the substrate surface, thereby allowing characterization of out‐of‐plane stacking of the NC assemblies independently of their in‐plane structure. A highly ordered superlattice is composed of alternating layers of organic and inorganic layers, which can lead to secondary diffraction and cause the splitting of the Bragg peaks with the appearance of additional fringes. The fringes are especially relevant in lamellar face‐on assemblies of thin NPLs while they tend to disappear in the edge‐on configuration, therefore constituting a good figure of merit for the analysis of NPL films.^[^ ^150^, ^187^ ^]^ The analysis of multilayer diffraction fringes can be used to determine the superlattice periodicity and the stacking disorders from the fringe position and the bandwidth, respectively. The complementary technique, the grazing incidence small‐angle X‐ray scattering (GISAXS) can be used to characterize large spacings of superlattices.^[^ ^146^ ^]^

## 7 Summary and Outlook

In the past decade, the emergence of solution‐processed, defect‐tolerant perovskite semiconductors has generated considerable research to demonstrate high‐efficiency solar cells and LEDs. For PeLEDs, recent research focus has been devoted to the development of new strategies for further enhancement of η_int_ and η_out_. In terms of the synthesis of LHP semiconductors, numerous defect passivation and post‐synthetic treatment strategies have been explored to reach near‐unity η_PL_, which significantly facilitates the enhancement of η_int_. This review encompasses the up‐to‐date EL performance of LHPs with various light‐outcoupling strategies with specific emphasis on emission anisotropy, including the effects of the long‐range ordering of LHP NCs on the emission TDM orientation. We classified the techniques into two major categories, the extrinsic and intrinsic light out‐coupling techniques. Each strategy discussed here could improve the η_out_, with a collective goal of surpassing 50% η_ext_ threshold critical benchmark for the next‐generation PeLEDs.

In the extrinsic light‐outcoupling sections, we covered the MLAs, HSLs, textured substrate surfaces, and air‐side scattering structures. Both MLAs and HSLs are very effective in extracting more light to air, through redirecting the trapped photons from the substrate and waveguide modes. On the other hand, the textured substrates and high‐index nanostructures allow the scattering of light in multiple directions, effectively increasing the probability for light to escape from the device dielectric stack. The intrinsic light outcoupling section discusses: i) the refractive index modulation, morphologies, and thickness of EML, ii) internal waveguide engineering, iii) optical microcavity design, iv) plasmonic nanostructures, v) photon recycling, and (vi) horizontal emission TDM orientation. The intrinsic light outcoupling techniques could also substantially suppress the energy dissipation through the waveguide, absorption, and plasmonic modes. The importance of the optimization of EML thickness, morphology, and distance from the metal electrode should not be underestimated, as these device parameters directly influence the internal waveguiding, plasmonic characteristics, and the spatial distribution of the emission within the device stack. In general, a thinner EML and precise control over EML morphology reduce the waveguide losses, thus improving outcoupling efficiency. The employment of low‐index light‐emitting materials and auxiliary layers, HTL and ETL, could also play an important role in curtailing the optical losses at the dielectric interfaces.

The photon recycling and microcavity structures can be also considered as intrinsic techniques that enhance the efficiency of light emission within the device stack before reaching the outcoupling stage. Photon recycling involves the reabsorption and re‐emission of photons within the EML, effectively granting more opportunities for photons to be dissipated in the lossy modes. The microcavity structures, by carefully tuning the optical pathways, can induce constructive interference for the emitted light at specific wavelengths, thereby directing more photons into the air.

We consider the manipulation of the EML TDM orientation among the most promising methods to enhance outcoupling efficiencies of PeLEDs, particularly for those based on perovskite NCs. The anisotropic emission, where the EML ensemble TDM is horizontally oriented with respect to the substrate plane, can substantially boost η_out_ by up to 55%, directing more light within the critical angle of the substrate–air interface. The ensemble TDM orientation in LHP NC films involves several effects, such as shape anisotropy of NCs, dielectric contrast between the LHP NCs and capping ligands or surrounding medium, packing ordering of NCs in the thin films, and superlattice formation. For instance, LHP NCs with anisotropic shapes, such as NPLs, tend to have TDMs aligned along their long axis, which can be used to align TDM in ensemble films. The capping ligands also influence the TDM orientation by increasing the dielectric contrast, surface electric field, and aspect ratio of LHP NCs. The crystal structure of the LHPs, notably the degree of octahedral tilting and the nature of the organic cation, influences the TDM orientation through the long‐range ordering of NCs that results in superlattice assemblies, making it an important aspect in engineering high‐efficiency devices. The TDM is influenced by the packing ordering of anisotropic NCs. Notably, the randomly oriented anisotropic LHP NCs exhibit a lower Θ_IP_ value than those of highly ordered face‐on superlattices.

Considering the current development of LHP materials on the ultimate stoichiometric stability, long‐range ordering, and effective defect passivation of LHP NPLs, we anticipate a new series of perovskite emitters can be obtained by varying the anion composition to cover the entire visible region. Chemical engineering these emitters may not only boost the η_out_ but also enhance the η_PL_ due strong quantum confinement effect and exciton binding energy. The emission chromaticity of the LHP NPLs can be tuned in the entire visible spectral range without compromising the intrinsic in‐plane TDM orientation and aspect ratio. Moreover, the LHP NPLs can form face‐on orientation assemblies upon simple spin coating.

We have comprehensively discussed a number of popular techniques for the characterization of emission anisotropy and the determination of TDM orientation of LHP EML thin films, including VASE, ADPL, BFP or *k*‐space (BFP) imaging, and GIWAXS. We have critically analyzed their strengths and limitations. Despite rather complicated instrumentation, we consider direct BFP imaging the most reliable method to quantitatively determine Θ_IP_. We anticipate the observations and highlights presented here will facilitate accurate determination of ensemble TDM orientation, which is increasingly important in this community.

Very recently, an active research area has been the development of synergetic light extraction approaches that take into account multiple effects and trade‐offs. Particularly, the combination of both extrinsic and intrinsic light outcoupling strategies may represent the solution to fully unlock the potential of PeLEDs. The conception of synergetic light outcoupling techniques should look into the following two factors: i) chemical and material properties of LHP EML and ii) complementary light outcoupling functions resulting from available techniques. For example, the high‐refractive‐index bulk LHP thick films with tailored surface morphologies are emerging as a promising candidate that enhance η_out_ through photon recycling. However, the LHP emission layer demands negligible stokes shift, strong reabsorption of emitted photons, nearly non‐extrinsic absorption of photons, and appropriate emission layer thickness. Note that upon increasing the emission layer thickness, which is desirable to enhance photon recycling, the η_out_ could experience catastrophic drops due to undesirable microcavity effects.

In principle, device η_out_ can exceed over 70% by combining high Θ_IP_, the low‐refractive‐index EML / transport layers, and external HSLs. The intrinsic low refractive index materials and Θ_IP_ effectively outcouple the photons from the waveguide and evanescent modes, while the extrinsic HSLs strongly extracted the photons from the substrate mode. With ongoing research and innovation in this field, the future of high‐efficiency, cost‐effective, and versatile optoelectronic devices appears exceptionally bright.

## Conflict of Interest

The authors declare no conflict of interest.
